# Dysregulation of pseudogene/lncRNA-hsa-miR-363-3p-SPOCK2 pathway fuels stage progression of ovarian cancer

**DOI:** 10.18632/aging.102538

**Published:** 2019-12-03

**Authors:** Weiyang Lou, Bisha Ding, Guansheng Zhong, Chengyong Du, Weimin Fan, Peifen Fu

**Affiliations:** 1 Department of Breast Surgery, The First Affiliated Hospital, College of Medicine, Zhejiang University, Zhejiang Province, Hangzhou 310003, China; 2 Program of Innovative Cancer Therapeutics, Division of Hepatobiliary and Pancreatic Surgery, Department of Surgery, First Affiliated Hospital, College of Medicine, Zhejiang University, Zhejiang Province, Hangzhou 310003, China; 3 Key Laboratory of Organ Transplantation, Zhejiang Province, Hangzhou 310003, China; 4 Key Laboratory of Combined Multi-organ Transplantation, Ministry of Public Health, Zhejiang Province, Hangzhou 310003, China

**Keywords:** ovarian cancer, miRNA, pseudogene, lncRNA, SPOCK2

## Abstract

Objective: Ovarian cancer is one of the most common and lethal cancer types in women. The molecular mechanism of ovarian cancer progression is still unclear.

Results: Here, we first reported that expression levels of three genes, GJB2, S100A2 and SPOCK2, were significantly higher in advanced stage than that in early stage of ovarian cancer, and upregulation of them indicated poor prognosis of patients with ovarian cancer. Subsequently, 8, 6 and 20 miRNAs were predicted to target GJB2, S100A2 and SPOCK2, respectively. Among these miRNA-mRNA pairs, hsa-miR-363-3p-SPOCK2 axis was the most potential in suppressing progression of ovarian cancer. Mechanistically, we found that hsa-miR-363-3p-SPOCK2 axis was involved in regulation of actin cytoskeleton. Moreover, 6 pseudogenes and 8 lncRNAs were identified to potentially inhibit hsa-miR-363-3p-SPOCK2 axis in ovarian cancer.

Conclusions: Collectively, we elucidate a regulatory role of pseudogene/lncRNA-hsa-miR-363-3p-SPOCK2 pathway in progression of ovarian cancer, which may provide effective therapeutic approaches and promising prognostic biomarkers for ovarian cancer.

Materials and methods: Differentially expressed genes (DEGs) in ovarian cancer were first screened using GSE12470, after which DEGs expression were validated using GEPIA. Kaplan-Meier analysis was employed to assess the prognostic values. Potential miRNAs were predicted by seven target prediction databases, and upstream lncRNAs and pseudogenes of hsa-miR-363-3p were forecasted through miRNet or starBase. UALCAN and starBase were used to obtain the co-expressed genes of SPOCK. Enrichment analysis for these co-expressed genes was performed by Enrichr.

## INTRODUCTION

It is known to all that RNAs consist of coding RNAs (messenger RNAs, mRNAs) and noncoding RNAs (ncRNAs). In recent years, ncRNAs have been the centerpiece of human genome research [1]. NcRNAs have key implications for human health and dysregulation of ncRNAs causes a variety of human disorders, including cancer [2, 3]. There are many types of ncRNAs, including microRNA (miRNA), long ncRNA (lncRNA), pseudogene and circular RNA (circRNA) [4, 5]. In 2011, the team of Salmena et al. proposed competing endogenous RNA (ceRNA) hypothesis, which is a regulatory mechanism between mRNAs and ncRNAs [6]. ceRNA mechanism demonstrates that lncRNAs, pseudogenes, circRNAs and mRNAs can cross-talk by competitively binding to shared miRNAs, thereby exerting their biological functions [7]. Increasing ncRNAs have been discovered to act as important tumor promoters or suppressors [8–11]. Moreover, ncRNAs also serve as potential diagnostic and prognostic biomarkers for cancer [12–15]. To date, only an extremely limited number of ncRNAs have been characterized functionally in cancer, though thousands of ncRNAs have been annotated.

Ovarian cancer ranks the seventh most common neoplasm among the world and the eighth leading cause of cancer deaths in women [16, 17]. Globally, 239,000 new patients were affected by ovarian cancer and 152,000 deaths occurred every year [18]. It’s widely known that ovarian cancer can be classified into three subtypes, including epithelial, specialized stromal cell and germ cell tumors, among which epithelial ovarian cancer occupies largest ratio in ovarian cancer. Besides, epithelial ovarian cancer is the most lethal gynecologic malignancy. Different types of epithelial ovarian cancer have distinct mutational spectrum and different prognosis [19–21]. To date, surgery and platinum/ taxane chemotherapy combinate as the main treatment for ovarian cancer [22, 23]. It’s an effective method for early stage ovarian cancer patients, with the five-year survival rates up to 92% [24]. However, in spite of huge advances in various therapies in the past few decades, only 19% ovarian cancer patients are diagnosed at early stage, due to deficiency of typical clinical symptoms and manifestations and fast tumor progression, and the five-year survival rates of ovarian cancer are still only below 30% [25]. Hence, it’s extremely meaningful to rapidly seek potential biomarkers and therapeutic targets for improving outcomes of ovarian cancer patients.

**Figure 1:**
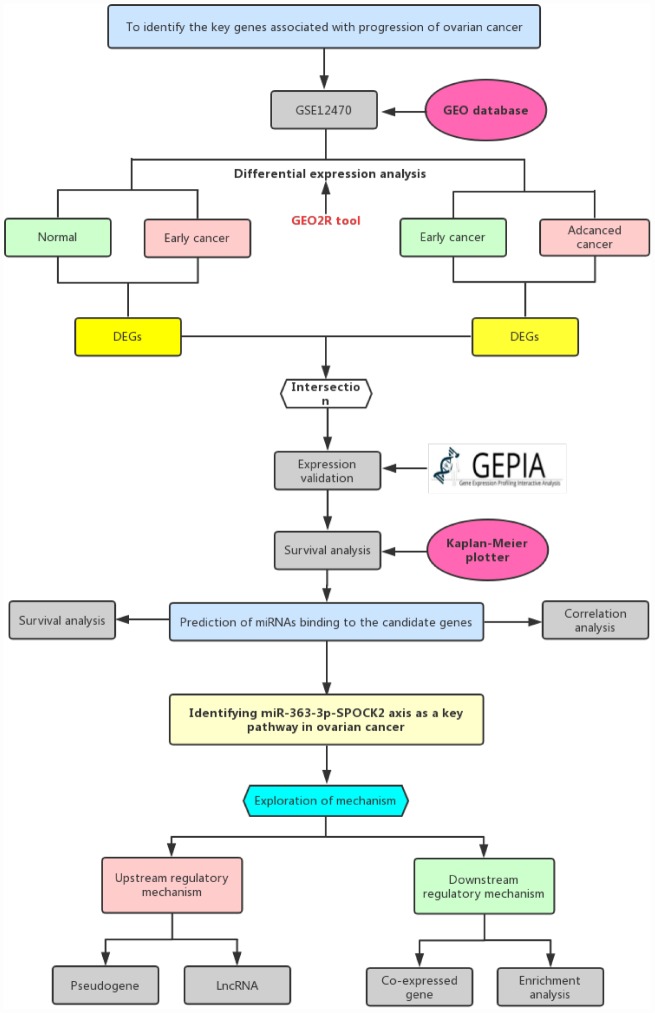
**The visual flow-process diagram of this study.**

In this study, we constructed a network linked to stage progression of ovarian cancer by a series of analytic processes (Figure 1). Firstly, we obtained differentially expressed genes (DEGs)-associated with stage progression of ovarian cancer using GEO dataset, GSE12470. Next, TCGA ovarian cancer cohort was employed to validate expression of these DEGs and perform survival analysis. Subsequently, upstream miRNAs of SPOCK2 were predicted. By expression correlation analysis and survival analysis, the most potential miRNA (hsa-miR-363-3p) binding to SPOCK2 was identified. Finally, the potential upstream dysregulated mechanisms (pseudogenes and lncRNAs) and downstream modulatory pathway were explored. The established pseudogene/lncRNA-hsa-miR-363-3p-SPOCK2 pathway shed novel insight into molecular mechanism of ovarian cancer progression and may provide effective therapeutic targets and promising prognostic biomarkers for ovarian cancer.

## RESULTS

### Screen of candidate genes associated with stage progression of ovarian cancer

**Figure 2:**
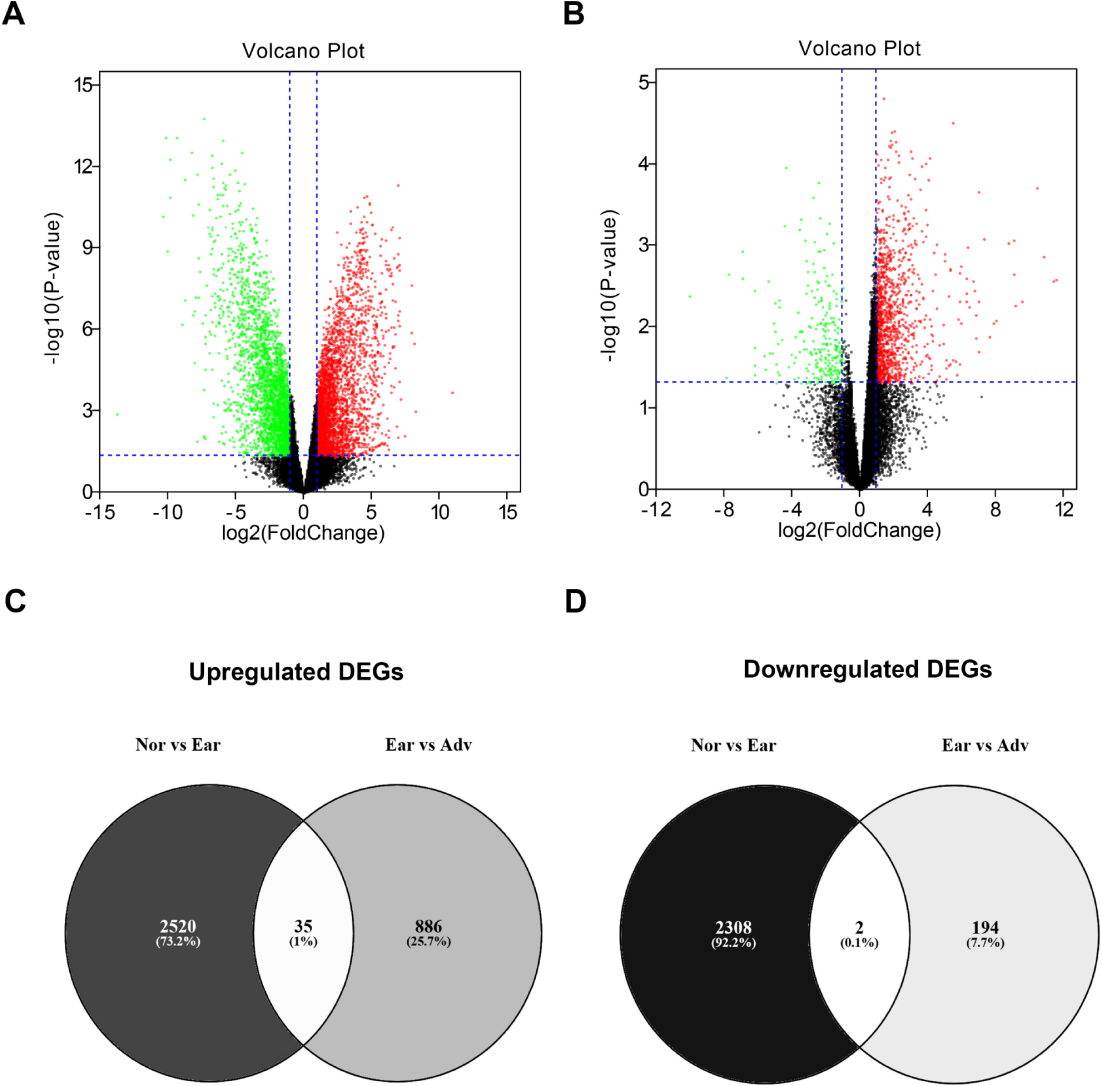
**Identification of differentially expressed genes (DEGs) between ovarian cancer and normal controls.** (**A**) Volcano plot showing the DEGs between normal samples (n=10) and early ovarian cancer (n=8). (**B**) Volcano plot showing the DEGs between early ovarian cancer (n=8) and advanced ovarian cancer (n=35). Note: the black dots represent genes that are not significantly differentially expressed between two groups, and the green dots and red dots represent the downregulated and upregulated genes in early ovarian cancer (compared with normal samples) and advanced ovarian cancer (compared with early ovarian cancer), respectively. |log_2_FC| > 1 and P-value < 0.05 were set as the cut-off criteria. FC = fold change. (**C**) The intersection of upregulated DEGs of “Nor vs Ear” and “Ear vs Adv”. (**D**) The intersection of downregulated DEGs of “Nor vs Ear” and “Ear vs Adv”. “Nor” vs Ear” represents the differential expression analysis between normal samples and early ovarian cancer. “Ear vs Adv” represents the differential expression analysis between early ovarian cancer and advanced ovarian cancer.

To identify the key genes involved in stage progression of ovarian cancer, GSE12470 dataset was selected to perform differential expression analysis using GEO2R. As described in materials and methods, all cases of GSE12470 were divided into three groups: “Nor” group, “Ear” group and “Adv” group. A lot of differentially expressed genes (DEGs) between “Nor” group and “Adv” group or between “Ear” group and “Adv” group were discovered as shown in Figure 2A and Figure 2B, respectively. A total of 2,555 and 2,310 DEGs were significantly upregulated and downregulated in early ovarian cancer samples compared with normal samples (Supplementary Table 1), respectively. And a total of 921 and 196 DEGs were significantly upregulated and downregulated in advanced ovarian cancer samples compared with early ovarian cancer samples, respectively (Supplementary Table 2). This study aims to find those genes associated with stage progression of ovarian cancer. Therefore, we obtained the upregulated and downregulated DEGs that were commonly appeared in two comparison sets. As presented in Figure 2C–D and Table 1, 35 upregulated DEGs and 2 downregulated DEGs were finally identified. The 37 DEGs were defined as candidate genes and selected for subsequent analysis.

**Table 1:** Expression change of 37 significant candidate gene in ovarian cancer.

**Gene symbol**	**Log_2_FC^a^**	**Log_2_FC^b^**
ADAMDEC1	2.76	1.95
C11orf45	1.59	1.30
CCNA2	4.10	1.34
CHEK1	2.88	1.37
CLGN	3.32	1.81
DAPL1	5.59	2.17
EGFL6	2.68	1.79
FAM122B	1.30	1.13
FAM181A	1.70	1.30
FAM83D	2.46	1.10
FOXA2	3.19	1.57
FRAS1	2.83	2.02
GJB2	3.04	2.85
GMCL1	1.44	1.02
GPR19	3.20	1.32
HRK	2.22	1.53
HTR2C	2.61	3.12
L3MBTL1	2.33	1.39
LCT	5.25	6.82
LRRTM1	3.22	2.55
LYPD1	3.19	1.56
MAD2L1	3.01	1.05
NEIL3	1.98	1.10
PART1	3.13	1.92
PNOC	3.96	2.97
RBM11	1.65	1.01
S100A2	1.85	1.43
SPOCK2	1.02	1.53
ST6GALNAC2	1.42	1.59
TGM1	2.12	2.07
TMPO	1.18	1.09
TMPRSS6	1.91	2.94
XPR1	1.05	1.38
ZBED2	5.98	1.53
ZNF556	2.46	1.17
EPS8	-1.48	-1.17
FAXDC2	-2.23	-2.30

^a^FC = (Early ovarian cancer samples)/(Normal samples);

^b^FC = (Advanced ovarian cancer samples)/(Early ovarian cancer samples).

### Expression validation and survival analysis of candidate genes in ovarian cancer

**Figure 3:**
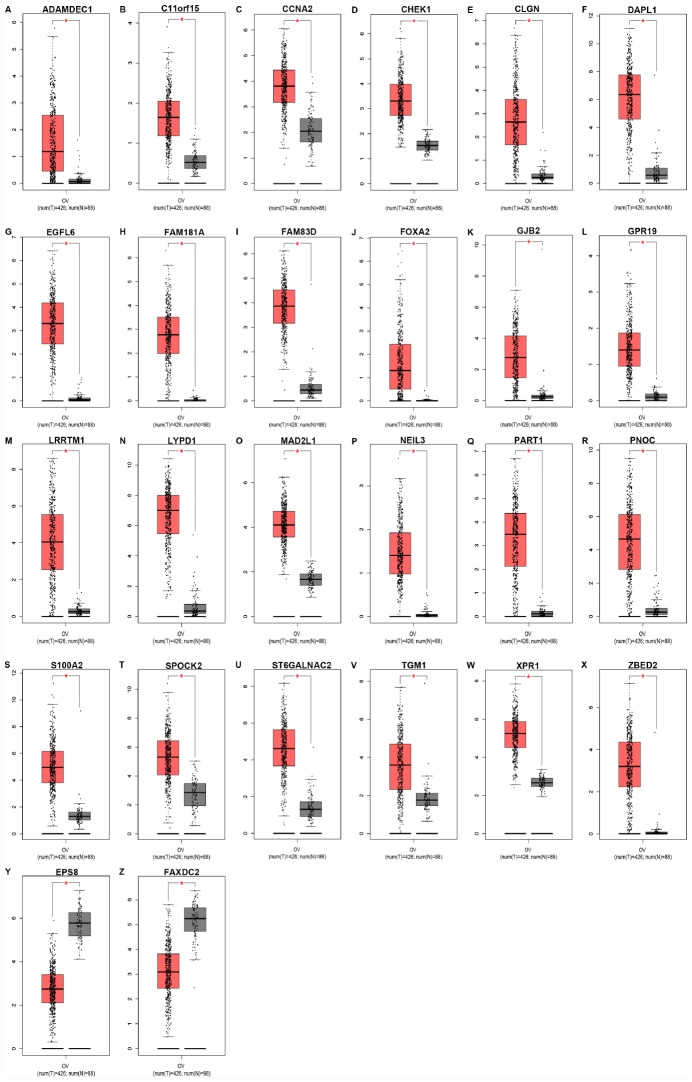
**Expression levels of 26 key genes** (ADAMDEC1 (**A**), C11orf15 (**B**), CCNA2 (**C**), CHEK1 (**D**), CLGN (**E**), DAPL1 (**F**), EGFL6 (**G**), FAM181A (**H**), FAM83D (**I**), FOXA2 (**J**), GJB2 (**K**), GPR19 (**L**), LRRTM1 (**M**), LYPD1 (**N**), MAD2L1 (**O**), NEIL3 (**P**), PART1 (**Q**), PNOC (**R**), S100A2 (**S**), SPOCK2 (**T**), ST6GALNAC2 (**U**), TGM1 (**V**), XPR1 (**W**), ZBED2 (**X**), EPS8 (**Y**) and FAXDC2 (**Z**)) in ovarian cancer determined by GEPIA database. “*” represents “P-value < 0.05”. Y axis indicates relative expression value, log_2_(TPM+1). TPM=Transcript per million.

**Figure 4:**
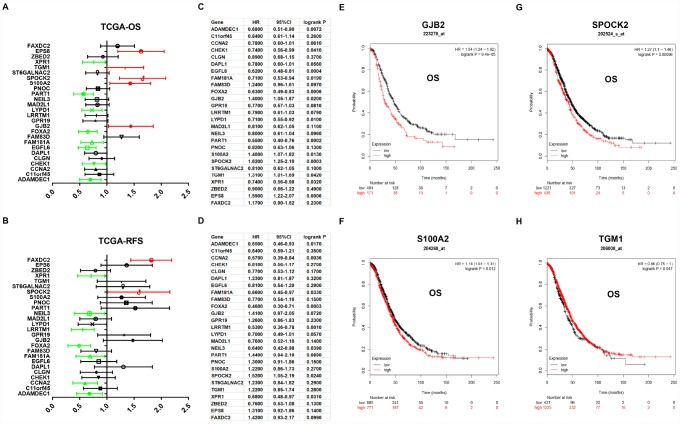
**Prognostic values of 26 candidate genes in ovarian cancer assessed by Kaplan-Meier plotter database.** (**A**) Prognostic values (overall survival, OS) of the 26 key genes in TCGA ovarian cancer cohort. (**B**) Prognostic values (relapse free survival, RFS) of the 26 key genes in TCGA ovarian cancer cohort. Green bars indicate a favorable prognosis; red bars indicate an unfavorable prognosis; black bars represent no statistical significance. (**C**) The detailed information of (**A**). (**D**) The detailed information of (**B**). (**E**) The prognostic value of GJB2 in 223278_at ovarian cancer cohort. (**F**) The prognostic value of S100A2 in 204268_at ovarian cancer cohort. (**G**) The prognostic value of SPOCK2 in 202524_s_at ovarian cancer cohort. (**H**) The prognostic value of TGM1 in 206008_at ovarian cancer cohort. Logrank P < 0.05 was considered as statistically significant.

Next, for improving accuracy of analysis, TCGA ovarian cancer samples and GTEx normal data were introduced to validate expression levels of 37 candidate genes *via* GEPIA database. As shown in Figure 3, among the 37 candidate genes, expression of 26 genes (ADAMDEC1, C11orf15, CCNA2, CHEK1, CLGN, DAPL1, EGFL6, FAM181A, FAM83D, FOXA2, GJB2, GPR19, LRRTM1, LYPD1, MAD2L1, NEIL3, PART1, PNOC, S100A2, SPOCK2, ST6GALNAC2, TGM1, XPR1, ZBED2, EPS8 and FAXDC2) were in accordance with the results acquired from GSE12470. Notably, only two genes (EPS8 and FAXDC2) were downregulated in cancer tissues compared with normal controls. In the following survival analysis, we focused on the 26 candidate genes. Firstly, TCGA ovarian cancer cohort was introduced to assess the prognostic values (including OS and RFS) of the 26 candidate genes. As shown in Figure 4A, high expression of GJB2, S100A2, SPOCK2, TGM1or EPS8 indicated a poor OS of patients with ovarian cancer, whereas ovarian cancer patients with higher expression of ADAMDEC1, CHEK1, EGFL6, FAM181A, FOXA2, LYPD1, PART1 or XPR1 possessed better OS. For RFS of patients with ovarian cancer, SPOCK2 and FAXDC2 were unfavorable prognostic biomarkers but ADAMDEC1, CCNA2, FAM181A, FOXA2, LRRTM1, NEIL3 and XPR1 were favorable prognostic biomarkers (Figure 4B). Among these genes, only expression levels of GJB2, S100A2, SPOCK2 and TGM1 were significantly upregulated in cancer samples and their high expression indicated poor prognosis (OS or RFS). The detailed information of survival analysis was listed in Figure 4C and Figure 4D. Subsequently, we introduced the ovarian cancer data other than TCGA to further assess the survival effects of GJB2, S100A2, SPOCK2 and TGM1 on prognosis (OS) by Kaplan-Meier plotter. As presented in Figure 4E–4G, ovarian cancer patients with higher expression of GJB2, S100A2 and SPOCK2 showed better OS, which was identical with the analytic results obtained from TCGA cohort. However, in 206008_at cohort, increased expression of TGM1 linked to favorable OS of ovarian cancer (Figure 4H), contrary to TCGA cohort. Taken together, GJB2, S100A2 and SPOCK2 might be three key genes in mediating tumor stage progression of ovarian cancer and causing poor prognosis.

### hsa-miR-363-3p-SPOCK2 axis is identified as a potential pathway linked to ovarian cancer progression

**Figure 5:**
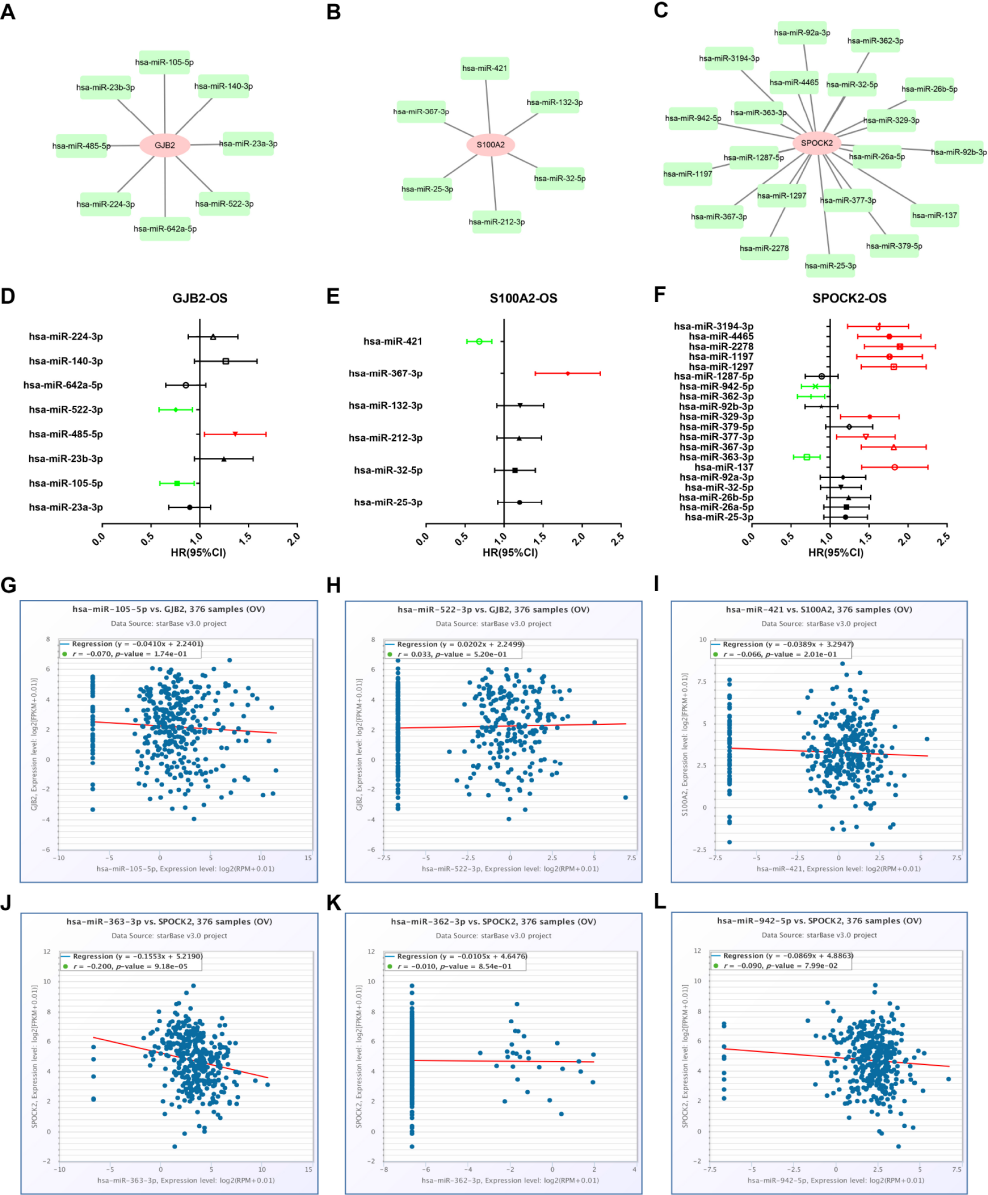
**Identification of upstream potential miRNAs of GJB2, S100A2 and SPOCK2 by combination of miRNA prediction, survival analysis and correlation analysis.** (**A**) The miRNA-GJB2 network established by Cytoscape. (**B**) The miRNA-S100A2 network established by Cytoscape. (**C**) The miRNA-SPOCK2 network established by Cytoscape. (**D**) Prognostic values (overall survival, OS) of potential upstream miRNAs of GJB2 in TCGA ovarian cancer cohort. (**E**) Prognostic values (overall survival, OS) of potential upstream miRNAs of S100A2 in TCGA ovarian cancer cohort. (**F**) Prognostic values (overall survival, OS) of potential upstream miRNAs of SPOCK2 in TCGA ovarian cancer cohort. Green bars indicate a favorable prognosis; red bars indicate an unfavorable prognosis; black bars represent no statistical significance. (**G**) The expression correlation of hsa-miR-105-5p and GJB2 in ovarian cancer. (**H**) The expression correlation of hsa-miR-522-3p and GJB2 in ovarian cancer. (**I**) The expression correlation of hsa-miR-421 and S100A2 in ovarian cancer. (**J**) The expression correlation of hsa-miR-363-3p and SPOCK2 in ovarian cancer. (**K**) The expression correlation of hsa-miR-362-3p and SPOCK2 in ovarian cancer. (**L**) The expression correlation of hsa-miR-942-5p and SPOCK2 in ovarian cancer.

Growing evidences have suggested that miRNAs mainly participate in negatively regulating gene expression and thereby play important roles in a variety of human biological processes, including cancer initiation and development [4, 26, 27]. Thus, we first predicted upstream miRNAs of GJB2, S100A2 and SPOCK2 through seven predicting programs as mentioned above (Table 2). Finally, 8, 6 and 20 upstream miRNAs were found to potentially target GJB2, S100A2 and SPOCK2, respectively. For better visualization, miRNA-GJB2, miRNA-S100A2 and miRNA-SPOCK2 sub-networks were established as shown in Figure 5A–5C. Then, we evaluated the prognostic values of these miRNAs in ovarian cancer using TCGA data. As shown in Figure 5D, among all the predicted miRNAs of GJB2, high expression of hsa-miR-522-3p and hsa-miR-105-5p indicated favorable OS of patients with ovarian cancer but hsa-miR-485-5p was an unfavorable prognostic biomarker for ovarian cancer. For S100A2, upregulation of hsa-miR-421 and hsa-miR-367-3p correlated with good and bad prognosis, respectively (Figure 5E). As presented in Figure 5F, regarding to these miRNAs of SPOCK2, high expression of hsa-miR-363-3p, hsa-miR-362-3p and hsa-miR-942-5p showed favorable OS but increased expression of hsa-miR-137, hsa-miR-367-3p, hsa-miR-377-3p, hsa-miR-329-3p, hsa-miR-1297, hsa-miR-1197, hsa-miR-2278, hsa-miR-4465 and hsa-miR-3194-3p linked to unfavorable OS. In view of miRNA functional mechanism and oncogenic roles of GJB2, S100A2 and SPOCK2, upstream miRNAs of the three genes should be tumor suppressive miRNAs. Therefore, six miRNA-mRNA pairs, including hsa-miR-105-5p-GJB2, hsa-miR-522-3p-GJB2, hsa-miR-421-S100A2, hsa-miR-363-3p-SPOCK2, hsa-miR-362-3p-SPOCK2 and hsa-miR-942-5p-SPOCK2, were selected for subsequent expression correlation analysis. Figure 5G–5L told us that only hsa-miR-363-3p was significantly negatively correlated with SPOCK2 in ovarian cancer. Altogether, hsa-miR-363-3p-SPOCK2 axis may be the most potential pathway in mediating tumor stage progression of ovarian cancer.

**Table 2:** Prediction of miRNAs binding to GJB2, S100A2 or SPOCK2.

**Gene name**	**miRNA name**	**Predicting program**	**Number**
GJB2	hsa-miR-23a-3p	PITA, miRanda	2
GJB2	hsa-miR-105-5p	PITA, miRmap, microT, PicTar	4
GJB2	hsa-miR-23b-3p	PITA, miRanda	2
GJB2	hsa-miR-485-5p	PITA, miRmap	2
GJB2	hsa-miR-522-3p	PITA, miRmap, microT	3
GJB2	hsa-miR-642a-5p	PITA, miRmap	2
GJB2	hsa-miR-140-3p	PITA, miRmap	2
GJB2	hsa-miR-224-3p	miRmap, microT	2
S100A2	hsa-miR-25-3p	RNA22, TargetScan	2
S100A2	hsa-miR-32-5p	RNA22, TargetScan	2
S100A2	hsa-miR-212-3p	PITA, miRanda	2
S100A2	hsa-miR-132-3p	PITA, miRanda	2
S100A2	hsa-miR-367-3p	RNA22, TargetScan	2
S100A2	hsa-miR-421	PITA, miRmap, microT, miRanda	4
SPOCK2	hsa-miR-25-3p	PITA, microT, miRanda, TargetScan	4
SPOCK2	hsa-miR-26a-5p	PITA, RNA22, PicTar, TargetScan	4
SPOCK2	hsa-miR-26b-5p	PITA, RNA22, PicTar, TargetScan	4
SPOCK2	hsa-miR-32-5p	PITA, microT, miRanda, TargetScan	4
SPOCK2	hsa-miR-92a-3p	PITA, microT, miRanda, TargetScan	4
SPOCK2	hsa-miR-137	PITA, TargetScan	2
SPOCK2	hsa-miR-363-3p	PITA, microT, miRanda, TargetScan	4
SPOCK2	hsa-miR-367-3p	PITA, microT, miRanda, TargetScan	4
SPOCK2	hsa-miR-377-3p	PITA, miRmap, microT, miRanda	4
SPOCK2	hsa-miR-379-5p	PITA, miRmap	2
SPOCK2	hsa-miR-329-3p	PITA, microT	2
SPOCK2	hsa-miR-92b-3p	PITA, microT, miRanda, TargetScan	4
SPOCK2	hsa-miR-362-3p	PITA, microT	2
SPOCK2	hsa-miR-942-5p	PITA, miRmap	2
SPOCK2	hsa-miR-1287-5p	PITA, miRmap	2
SPOCK2	hsa-miR-1297	PITA, PicTar, TargetScan	3
SPOCK2	hsa-miR-1197	PITA, miRmap, microT	3
SPOCK2	hsa-miR-2278	miRmap, PicTar, RNA22	3
SPOCK2	hsa-miR-4465	RNA22, PicTar, TargetScan	3
SPOCK2	hsa-miR-3194-3p	miRmap, microT	2

### Co-expression and enrichment analyses reveal that hsa-miR-363-3p-SPOCK2 axis is involved in regulation of actin cytoskeleton

**Figure 6:**
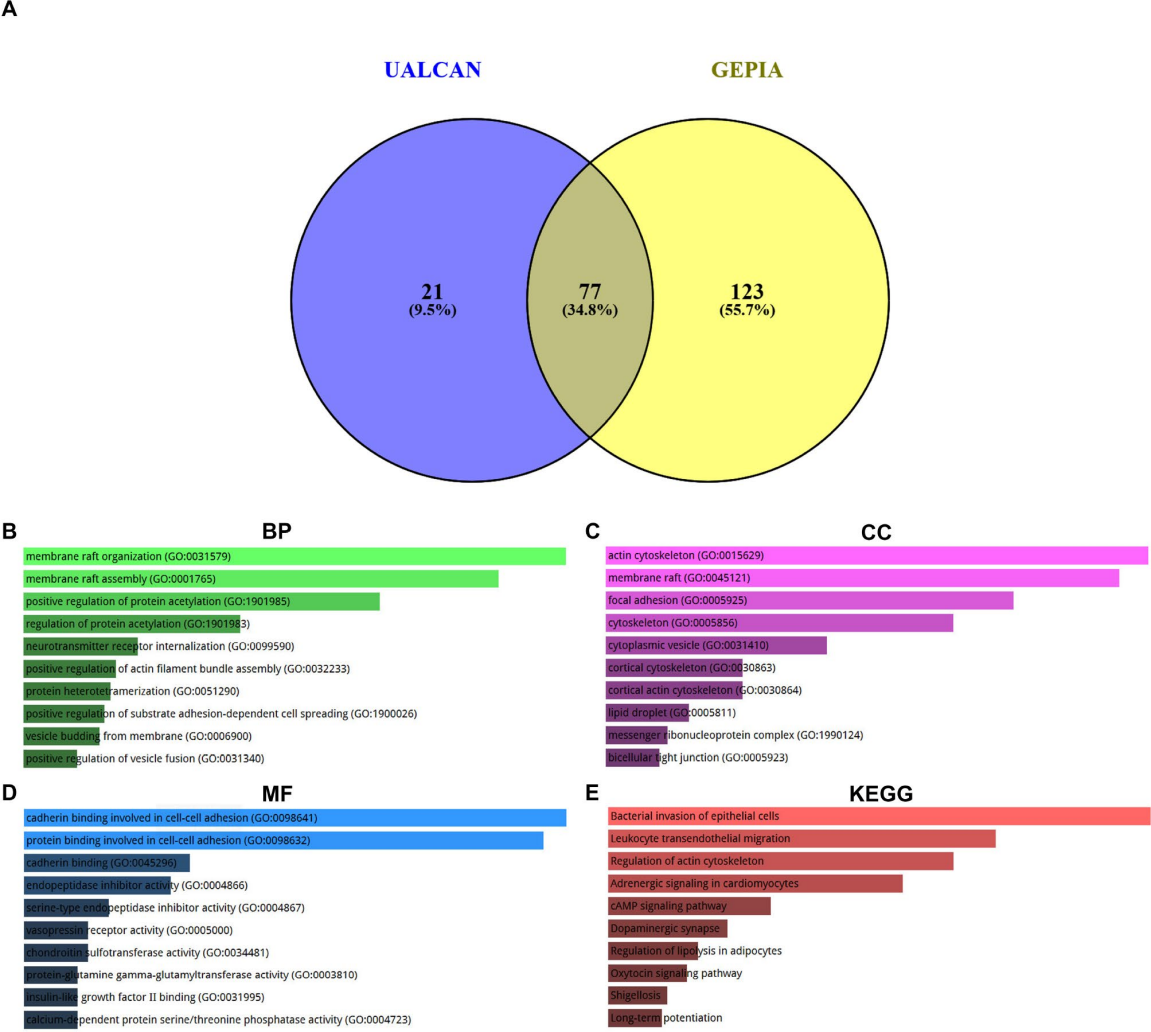
**Enrichment analysis of co-expressed genes of SPOCK2 in ovarian cancer.** (**A**) Identification of co-expressed genes of SPOCK2 in ovarian cancer using UALCAN and GEPIA database. (**B**) The top 10 enriched biological process (BP) items for the co-expressed genes of SPOCK2. (**C**) The top 10 enriched cellular component (CC) items for the co-expressed genes of SPOCK2. (**D**) The top 10 enriched molecular function (MF) items for the co-expressed items for the co-expressed genes of SPOCK2. (**E**) The top 10 enriched KEGG items for the co-expressed items for the co-expressed genes of SPOCK2.

Co-expression analysis was performed using two databases, namely UALCAN and GEPIA. 100 and 200 co-expressed genes were obtained from UALCAN and GEPIA as listed in Supplementary Table 3. By intersection of two gene sets, we found that 77 co-expressed genes of SPOCK2 were commonly appeared in the two databases (Figure 6A and Supplementary Table 4). The 77 genes and their correlation coefficients were presented in Table 3. GO functional annotation and KEGG pathway enrichment analysis were introduced to better understand these genes *via* Enrichr database. The top 10 enriched GO terms were shown in Figure 6B–6D, containing membrane raft organization, membrane raft assembly and positive regulation of protein acetylation in the BP category, actin cytoskeleton, membrane raft and focal adhesion in the CC category, and cadherin binding involved in cell-cell adhesion, protein binding involved in cell-cell adhesion and cadherin binding in the MF category. The top 10 enriched KEGG pathways were presented in Figure 6E, including bacterial invasion of epithelial cells, leukocyte transendothelial migration and regulation of actin cytoskeleton. These findings demonstrate that hsa-miR-363-3p-SPOCK2 axis may regulate actin cytoskeleton and thereby take part in cell adhesion, invasion and migration, and finally suppress progression of ovarian cancer.

**Table 3:** The co-expressed genes of SPOCK2 commonly appeared in UALCAN database and GEPIA database.

**Common co-expressed genes of SPOCK2**	**R^a^**	**R^b^**
RASGRP4	0.59	0.62
SLC48A1	0.57	0.58
ADORA1	0.53	0.50
UNC5B	0.53	0.58
OXTR	0.52	0.52
CRB2	0.52	0.55
TRIM36	0.50	0.47
CADM3	0.48	0.46
SLC22A18AS	0.48	0.41
ZBED2	0.47	0.53
TNS3	0.47	0.49
KIF21A	0.46	0.43
S100A10	0.45	0.43
CLIC5	0.45	0.44
CAMK2G	0.44	0.46
RAB19	0.42	0.46
CACNG4	0.42	0.41
TBC1D2	0.41	0.43
UPK3B	0.41	0.41
MYADM	0.41	0.42
ANXA7	0.40	0.39
CLDN15	0.40	0.40
WNT10A	0.40	0.41
CCDC85A	0.39	0.39
CD151	0.39	0.38
CASKIN2	0.39	0.37
AMOTL2	0.38	0.43
DTX4	0.38	0.40
IGFBP6	0.37	0.34
ANXA2P2	0.37	0.36
ST6GAL2	0.37	0.34
ANXA2	0.37	0.46
KRT80	0.37	0.39
CLSTN2	0.37	0.37
ANXA9	0.37	0.37
DNM3	0.37	0.41
SHISA4	0.36	0.37
PLA2G7	0.36	0.34
INF2	0.36	0.35
CHRDL1	0.36	0.42
RAPGEF3	0.36	0.40
SLC4A11	0.36	0.35
PNPLA2	0.36	0.32
RNASEL	0.35	0.36
TPRN	0.35	0.36
OLFML2A	0.35	0.35
TGM1	0.35	0.37
VSIG10L	0.35	0.32
SERPINB5	0.35	0.32
LRRN4	0.34	0.35
FNDC4	0.34	0.37
PRSS33	0.34	0.32
ST5	0.34	0.35
GNG12	0.33	0.33
VSTM2L	0.33	0.35
TNNT2	0.33	0.31
BAIAP2	0.33	0.37
ARL13B	0.32	0.37
SERPINA5	0.32	0.31
ZMIZ1	0.32	0.36
CHST11	0.32	0.34
SLC29A3	0.32	0.37
ANO9	0.32	0.31
PKP3	0.31	0.31
VCL	0.31	0.34
ARNTL	0.31	0.32
BET1L	0.31	0.35
TMEM9B	0.31	0.31
GDPD5	0.30	0.31
SCD5	0.30	0.33
FAM69A	0.30	0.31
TNS1	0.30	0.32
ARPC1B	0.30	0.31
SMPD1	0.30	0.32
PXN	0.30	0.35
RHOF	0.30	0.35
RIC8A	0.30	0.34

^a^Correlation coefficient determined by UALCAN database.

^b^Correlation coefficient determined by GEPIA database.

### Upstream potential pseudogenes and lncRNAs of hsa-miR-363-3p

**Figure 7:**
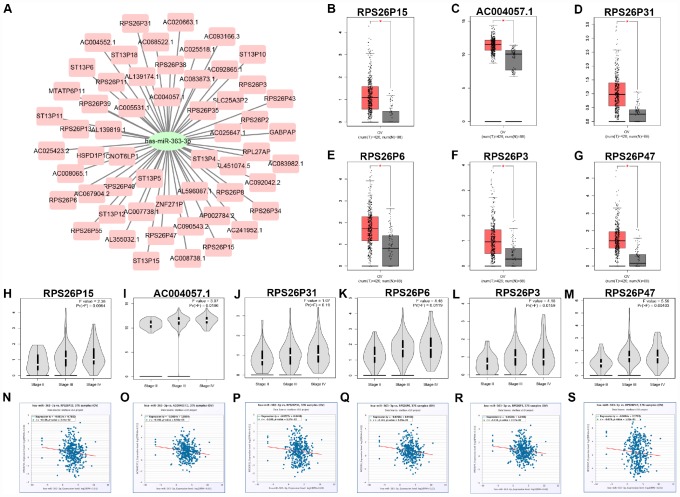
**Identification of upstream potential pseudogenes of hsa-miR-363-3p in ovarian cancer.** (**A**) The pseudogenes-hsa-miR-363-3p network constructed by Cytoscape. The expression levels of RPS26P15 (**B**), AC004057.1 (**C**), RPS26P31 (**D**), RPS26P6 (**E**), RPS26P3 (**F**) and RPS26P47 (**G**) in ovarian cancer compared with normal controls. “*” represents “P-value < 0.05”. Y axis indicates relative expression value, log_2_(TPM+1). TPM=Transcript per million. Expression differences of RPS26P15 (**H**), AC004057.1 (**I**), RPS26P31 (**J**), RPS26P6 (**K**), RPS26P3 (**L**) and RPS26P47 (**M**) among various major stage in ovarian cancer. P-value < 0.05 was considered as statistically significant. (**N**) The expression correlation of hsa-miR-363-3p and RPS26P15 in ovarian cancer. (**O**) The expression correlation of hsa-miR-363-3p and AC004057.1 in ovarian cancer. (**P**) The expression correlation of hsa-miR-363-3p and RPS26P31 in ovarian cancer. (**Q**) The expression correlation of hsa-miR-363-3p and RPS26P6 in ovarian cancer. (**R**) The expression correlation of hsa-miR-363-3p and RPS26P3 in ovarian cancer. (**S**) The expression correlation of hsa-miR-363-3p and RPS26P47 in ovarian cancer.

**Figure 8:**
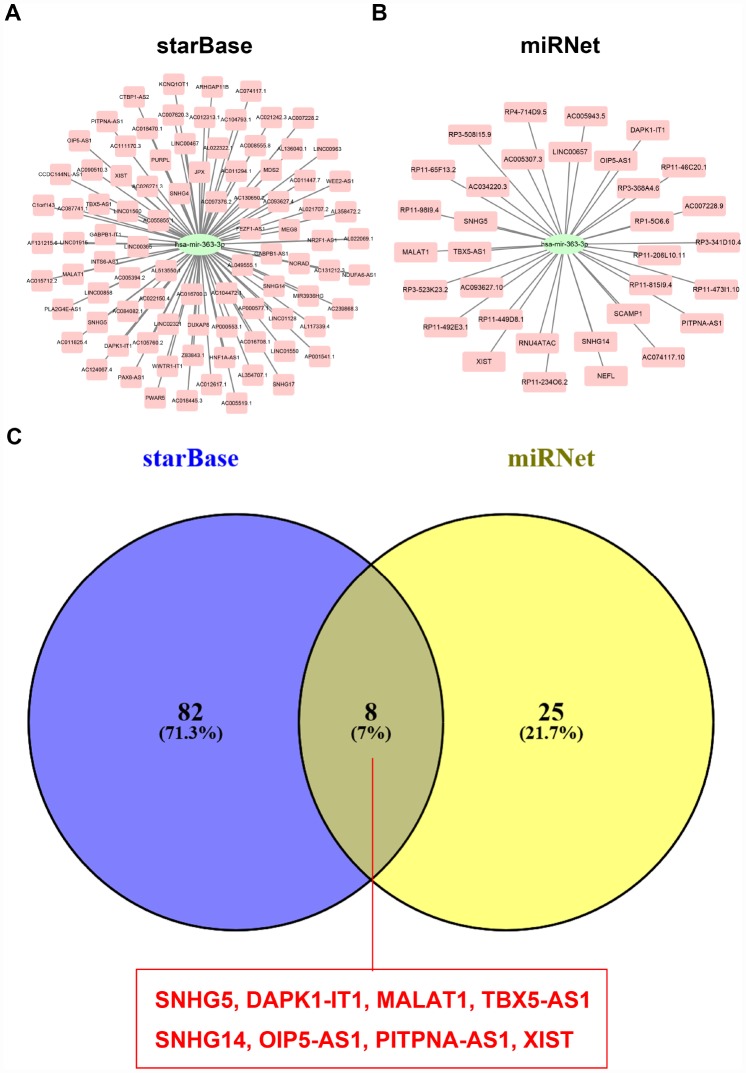
**Screening upstream potential lncRNAs of hsa-miR-363-3p.** (**A**) The potential lncRNAs of hsa-miR-363-3p predicted by starBase database. (**B**) The potential lncRNAs of hsa-miR-363-3p predicted by miRNet database. (**C**) 8 intersected lncRNAs (SNHG5, DAPK1-IT1, MALAT1, TBX5-AS1, SNHG14, OIP5-AS1, PITPNA-AS1 and XIST) from starBase and miRNet databases.

**Figure 9:**
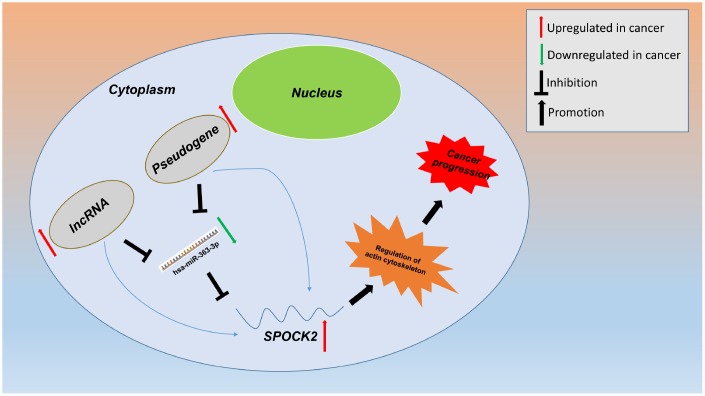
**Model of the pseudogene/lncRNA-hsa-miR-363-3p-SPOCK2 network and its expression and potential roles in ovarian cancer progression.**

Pseudogene and lncRNA are two considerable subtypes of noncoding RNA (ncRNA), which may function as ceRNAs to interact with mRNAs by competing for shared miRNAs. Therefore, starBase database was first utilized to predict the upstream potential pseudogenes that could potentially bind to hsa-miR-363-3p. 56 pseudogenes were obtained as shown in Figure 7A. Based on ceRNA mechanism, these pseudogenes should be oncogenes in ovarian cancer. The expression levels of the 56 pseudogenes were detected using GEPIA database. Finally, only 6 pseudogenes, RPS26P15 (Figure 7B), AC004057.1 (Figure 7C), RPS26P31 (Figure 7D), RPS26P6 (Figure 7E), RPS26P3 (Figure 7F) and RPS26P47 (Figure 7G,) were significantly upregulated in cancer tissues when compared with normal controls. Then, expression differences of the 6 pseudogenes among various major stages were also determined. As shown in Figure 7H–7M, all of the 6 pseudogenes expression were higher in advanced stage than that in early stage of ovarian cancer in general. Expression correlation analysis revealed that hsa-miR-363-3p was significantly correlated with RPS26P15 (Figure 7N), AC004057.1 (Figure 7O), RPS26P6 (Figure 7Q) or RPS26P3 (Figure 7R). For hsa-miR-363-3p-RPS26P31 (Figure 7P) and hsa-miR-363-3p-RPS26P47 (Figure 7S) pairs, no significant relationship was observed but still presented negative correlations. All these findings indicated that RPS26P15, AC004057.1, RPS26P31, RPS26P6, RPS26P3 and RPS26P47 were the most potential pseudogenes in regulating hsa-miR-363-3p in ovarian cancer. Subsequently, those lncRNAs that could potentially bind to hsa-miR-363-3p were also predicted. 90 and 33 upstream lncRNAs were found in starBase and miRNet as presented in Figure 8A and Figure 8B, respectively. The detailed lncRNAs were listed in Supplementary Table 5. By intersection of the two databases, eight lncRNAs (SNHG5, DAPK1-IT1, MALAT1, TBX5-AS1, SNHG14, OIP5-AS1, PITPNA-AS1 and XIST) were commonly appeared. Taken together, overexpressed lncRNAs/pseudogenes-mediated downregulation of hsa-miR-363-3p leads to increased expression of SPOCK2, modulating actin cytoskeleton and thereby resulting in progression of ovarian cancer (Figure 9).

## DISCUSSION

Ovarian cancer is notorious for its aggressive natural characteristic. Though great developments have been achieved in diagnosis and therapy for ovarian cancer, patients’ prognosis is still dismal. Exploration of molecular mechanisms of ovarian cancer progression is valuable and helpful for developing novel therapeutic approaches and thus improving patients’ survival.

In this study, based on comprehensive expression analysis and survival analysis, three genes (GJB2, S100A2 and SPOCK2) were identified as key genes which may be associated with progression of ovarian cancer. Expression levels of GJB2, S100A2 and SPOCK2 were increased in ovarian cancer and their upregulation was linked to poor prognosis of patients with ovarian cancer. The 3 genes have been confirmed to act as oncogenes in multiple human cancers and link to cancer progression. Besides, many studies have also demonstrated that the three genes may serve as promising biomarkers for cancer. For example, GJB2 promoted early stage breast cancer progression by regulating cancer stemness [28]; Zhu et al. suggested that GJB2 was a prognostic marker in patients with pancreatic cancer by genome-scale analysis [29]; the group of Sun D found that GJB2 was identified as predictive biomarkers for pancreatic cancer by integrated whole genome microarray analysis and immunohistochemical assay [30]; Masuda et al. indicated that overexpressed S100A2 was a prognostic marker for patients with stage II and III colorectal cancer [31]; Kwon et al. showed that S100A2 was significant for progression of prostate adenocarcinoma [32]. Regrading to SPOCK2, the group of Ren F confirmed that SPOCK2 contributed to the progression of endometrial cancer by modulating the biological behavior of cancer cells [33]. All these reports together with our current analytic results of expression and survival analysis demonstrate that GJB2, S100A2 and SPOCK2 may be three key oncogenes in the progression of ovarian cancer.

miRNAs, a class of identified ncRNA molecules, play important roles in regulating the biological behaviors by suppressing target gene expression [26, 27]. Therefore, we intended to seek those miRNAs targeting to GJB2, S100A2 or SPOCK2. Using several online databases, some potential miRNAs, including 8 for GJB2, 6 for S100A2 and 20 for SPOCK2, were predicted. According to the action mechanism of miRNA, these miRNAs should be tumor suppressive miRNAs in ovarian cancer. After performing survival analysis, six miRNA-mRNA pairs (hsa-miR-105-5p-GJB2, hsa-miR-522-3p-GJB2, hsa-miR-421-S100A2, hsa-miR-363-3p-SPOCK2, hsa-miR-362-3p-SPOCK2 and hsa-miR-942-5p-SPOCK2) possessed the most potential functions in ovarian cancer and were selected for subsequent expression correlation analysis. Correlation analysis showed that only hsa-miR-363-3p-SPOCK2 pair existed significant negative relationship. Altogether, hsa-miR-363-3p-SPOCK2 axis was considered as the potential pathway, involving in progression of ovarian cancer. Numerous studies supported that hsa-miR-363-3p functioned as a key suppressor in development of multiple human cancers. For example, hsa-miR-363-3p was found to inhibit tumor growth and metastasis of colorectal cancer via targeting SPHK2 [34]; He et al. suggested that hsa-miR-363-3p acted as a tumor suppressor in osteosarcoma cells by inhibiting PDZD2 [35]. These reports partially supported the accuracy of our bioinformatics analysis. Next, the co-expressed genes of SPOCK2 were acquired using UALCAN and GEPIA databases. Enrichment analysis for these co-expressed genes revealed that they were significantly enriched in actin cytoskeleton-associated pathways. It has been well documented that actin cytoskeleton is closely correlated with cancer invasion and metastasis [36, 37]. Hence, hsa-miR-363-3p-SPOCK2 axis may suppress invasion and metastasis through regulation of actin cytoskeleton and finally block stage progression of ovarian cancer.

In additional to miRNAs, there are also some other types of ncRNAs, such as lncRNAs and pseudogenes. They can serve as ceRNAs of mRNAs by competitively binding to common miRNAs, thereby participating in human health and disorders, containing cancer [13, 38, 39]. The upstream pseudogenes of hsa-miR-363-3p-SPOCK2 axis were first obtained by starBase database. Expression differences between ovarian cancer samples and control normal or among various major stages were further determined using GEPIA database. Among 56 predicted pseudogenes, only 6 pseudogenes (RPS26P15, AC004057.1, RPS26P31, RPS26P6, RPS26P3 and RPS26P47) were significantly upregulated in cancer samples (compared to normal samples) and advanced stage of ovarian cancer (compared to early stage ovarian cancer). Expression correlation analysis demonstrated that hsa-miR-363-3p was inversely linked to RPS26P15, AC004057.1, RPS26P31, RPS26P6, RPS26P3 or RPS26P47. By combination of ceRNA mechanism and these analytic findings, the six pseudogenes were identified as the potential regulators of hsa-miR-363-3p-SPOCK2 axis in ovarian cancer. Finally, using starBase and miRNet databases, the upstream regulatory lncRNAs of hsa-miR-363-3p-SPOCK2 axis were also predicted. Eight lncRNAs (SNHG5, DAPK1-IT1, MALAT1, TBX5-AS1, SNHG14, OIP5-AS1, PITPNA-AS1 and XIST) were commonly appeared in the two databases. Among these LncRNAs, MALAT1 and SNHG14 have been demonstrated to function as oncogenes in ovarian cancer. For example, lncRNA MALAT1 promoted proliferation and metastasis of epithelial ovarian cancer via the PI3K-AKT pathway [40]; lncRNA SNHG14 enhanced proliferation and metastasis of ovarian cancer by sponging miR-219a-5p [40]. The rest lncRNAs were also found to function as oncogenes in other types of human cancer. For example, lncRNA SNHG5 facilitated growth and invasion of melanoma by regulating the miR-26a-5p/TRPC3 pathway [41]; lncRNA OIP5-AS1 predicted poor prognosis and regulated cell proliferation and apoptosis in bladder cancer [42]; lncRNA XIST enhanced pancreatic cancer cells invasion through promotion of TGF-β2 expression by targeting miR-141-3p [43]. These reports further supported that the 8 lncRNAs, similar to 6 potential pseudogenes, may also play crucial roles in regulating hsa-miR-363-3p-SPOCK2 axis, thus involving in progression of ovarian cancer.

## CONCLUSIONS

In summary, a series of integrated bioinformatics analyses suggest that hsa-miR-363-3p-SPOCK2 axis may play key roles in progression of ovarian cancer by regulating actin cytoskeleton. Furthermore, the potential upstream pseudogenes and lncRNAs of has-miR-363-3p-SPOCK2 axis are successfully identified. The constitutes in this pseudogenes/lncRNAs-hsa-miR-363-3p-SPOCK2 network may be utilized as promising therapeutic targets and prognostic biomarkers in the future.

## MATERIALS AND METHODS

### Microarray

The mRNA microarray profiles of ovarian cancer were downloaded from GEO database (http://www.ncbi.nlm.nih.gov/geo) by searching keywords ((“gene” OR “mRNA” [all fields] AND (“ovarian cancer” OR “ovarian carcinoma” OR “EOC” [all fields]) AND “Homo sapiens” [porgn]). Then, the titles and abstracts of datasets were screened, and the full information of the datasets of interest were further evaluated and finally selected according to the following inclusion criteria: (1) selected datasets should be mRNA transcriptome data; (2) the data should be derived from tumor tissues and normal tissues of patients with ovarian cancer; (3) only datasets containing more than 5 cancer samples and 5 normal samples were included; (4) cancer samples in selected datasets should contain early stage and advanced stage ovarian cancer. Finally, only one dataset GSE12470 was included in this study. GSE12470 dataset, which is based on the platform of Agilent-012097 Human 1A Microarray (V2) G4110B, contained 53 tissue samples, involving 10 normal samples, 8 early ovarian cancer samples and 35 advanced ovarian cancer samples.

### Differential expression analysis

The differentially expressed genes (DEGs) were obtained by conducting differential expression analysis. GEO2R (http://www.ncbi.nlm.nih.gov/geo/geo2r), an online analytic tool provided by the GEO database, was employed to perform differential expression analysis. Firstly, we divided the 53 samples into three groups, namely normal group (“Nor”, 10 samples), early stage ovarian cancer group (“Ear”, 8 samples) and advanced stage ovarian cancer group (“Adv”, 35 samples). Then, differential expression analysis was successively done between “Nor” group and “Ear” group, “Ear” group and “Adv” group. |log_2_FC| > 1 and P-value < 0.05 were set as the cut-off criteria. Finally, VENNY 2.1.0 (http://bioinfogp.cnb.csic.es/tools/venny/index.html) was utilized to draw the Veen diagrams. The DEGs were commonly upregulated or downregulated in “Ear” group (compared with “Nor” group) and “Adv” group (compared with “Ear” group). These DEGs were re-defined as significant DEGs. FC = fold change.

### Gene Expression Profiling Interactive Analysis (GEPIA) database analysis

GEPIA database (http://gepia.cancer-pku.cn/detail.php), a newly developed interactive web server for analyzing the RNA sequencing expression data, included 9,736 tumors and 8,587 normal samples from The Cancer Genome Atlas (TCGA) and The Genotype-Tissue Expression (GTEx) projects [15, 44]. GEPIA database was used to validate the expression levels of significant DEGs in ovarian cancer. |log_2_FC| > 1 and P-value < 0.05 were set as the cut-off criteria. After entering the gene symbols into the “Gene” column, the expression box plots were automatically generated on the webpage. Similarly, gene expression differences among various major stages were also determined using GEPIA database. Besides, GEPIA database was also introduced to identify the co-expressed genes of SPOCK2 in ovarian cancer.

### Kaplan-Meier plotter analysis

Kaplan-Meier plotter is a database that can be capable of assess the effect of 54,000 genes on survival in 21 cancer types, and the miRNA subsystems in this database include 11,000 samples from 20 different cancer types [45, 46]. Kaplan-Meier plotter was utilized to evaluate the prognostic values of genes or microRNAs in ovarian cancer. Briefly, genes or microRNAs were first typed into the database. All cases of ovarian cancer were classified into a low expression group and a high expression group based on the median expression value. Subsequently, Kaplan-Meier survival plots were generated on the webpage. The hazard ratio (HR), 95% confidence interval (CI) and logrank P-value were also automatically calculated and displayed on the webpage. A logrank P-value < 0.05 was considered as statistically significant.

### miRNA prediction

In this study, a relatively comprehensive method of miRNA prediction was utilized. Seven databases, containing PITA (https://genie.weizmann.ac.il/pubs/mir07/mir07_dyn_data.html), RNA22 (https://cm. jefferson.edu/rna22/), miRmap (https://mirmap.ezlab.org/), microT (http://www.microrna.gr/webServer), miRanda (http://www.microrna.org/microrna/home.do), PicTar (http://www.pictar.org/) and TargetScan (www.targetscan.org/), were employed to predict the upstream miRNAs potentially binding to GJB2, S100A2 and SPOCK2. Only these miRNAs appeared more than one predicting programs were selected for subsequent analysis.

### starBase database analysis

starBase database is a widely-used open-source platform for studying the ncRNA interactions from CLIP-seq, degradome-seq and RNA-RNA interactome data [47, 48]. Herein, starBase database was introduced to analyze the expression correlation between miRNA and gene or pseudogene. R < -0.1 and P-value < 0.05 were set as the cut-off criteria for identifying the significant miRNA-gene/pseudogene pairs. starBase database was also used to predict the pseudogenes and lncRNAs that can theoretically bind to hsa-miR-363-3p.

### UALCAN database analysis

UALCAN database is a portal for analyzing gene expression and survival effect, which provides easy access to publicly available cancer transcriptome data including ovarian cancer [49, 50]. In this study, the database was utilized to obtain the co-expressed genes of SPOCK2 in ovarian cancer. Then, these co-expressed genes were intersected with the co-expressed genes acquired from GEPIA database as mentioned above. The co-expressed genes commonly appeared in the two databases were re-defined as co-expressed genes of interest and were chosen for subsequent enrichment analysis.

### Enrichr database analysis

Gene Ontology (GO) functional annotation and KEGG pathway enrichment analysis for the co-expressed genes of SPOCK2 was performed using Enrichr database as we previously described [51–53]. Three categories, including biological process (BP), cellular component (CC) and molecular function (MF), were included in GO functional annotation. The top 10 enriched GO items and KEGG pathways were displayed on the webpage and downloaded as images.

### miRNet database analysis

miRNet (https://www.mirnet.ca/), an easy-to-use comprehensive platform integrated data from several miRNA-linked databases (TarBase, miRTarBase, miRecords, miRanda), was employed to predict the potential lncRNAs binding to hsa-miR-363-3p. Subsequently, these lncRNAs were intersected with the lncRNAs obtained from starBase database to acquire the most potential regulatory lncRNAs of hsa-miR-363-3p.

## Supplementary Material

Supplementary Table 1

Supplementary Table 2

Supplementary Table 3

Supplementary Tables 4 and 5
