# Supplementary material for: Dysregulation of pseudogene/lncRNA-hsa-miR-363-3p-SPOCK2 pathway fuels stage progression of ovarian cancer
Source: Aging (Albany NY). 2019 Dec 3;11(23):11416–39. doi: 10.18632/aging.102538 (PMC6932902; doi:10.18632/aging.102538)
Supplement: Supplementary Table 1 [file aging-11-102538-s004..docx]

**Supplementary Table 1.** **The significant DEGs between normal samples and early ovarian cancer samples.**

| Upregulated DEGs in early ovarian cancer samples | Downregulated DEGs in early ovarian cancer samples |
| --- | --- |
| AAAS | A1BG |
| AACS | A2M |
| AADAC | A4GNT |
| ABAT | AADACL4 |
| ABCA1 | AAED1 |
| ABCA12 | AASS |
| ABCB11 | ABCA10 |
| ABCB8 | ABCA6 |
| ABCC4 | ABCA8 |
| ABCC6 | ABCA9 |
| ABCF3 | ABCB1 |
| ABCG5 | ABCC8 |
| ABHD11 | ABCG2 |
| ABHD12 | ABHD17B |
| ABHD16A | ABHD6 |
| ABHD17C | ABI3 |
| ABHD3 | ABI3BP |
| ABI2 | ABL1 |
| ACBD5 | ABL2 |
| ACE2 | ABLIM2 |
| ACKR2 | ABLIM3 |
| ACOT11 | ABR |
| ACOT4 | ABRA |
| ACOT8 | ABTB2 |
| ACP6 | ACACB |
| ACSF2 | ACADL |
| ACSM3 | ACAN |
| ACTL10 | ACAP3 |
| ACTL6A | ACAT1 |
| ACTR3B | ACKR1 |
| ACVR2B | ACKR3 |
| ACY1 | ACKR4 |
| ADAM10 | ACOT2 |
| ADAM28 | ACOX2 |
| ADAM30 | ACSL1 |
| ADAMDEC1 | ACSL4 |
| ADAMTS13 | ACSM2B |
| ADAP1 | ACSM5 |
| ADAR | ACSS3 |
| ADAT3 | ACTA2 |
| ADCK5 | ACTC1 |
| ADCY8 | ACTG2 |
| ADGRF1 | ACTL6B |
| ADGRG1 | ACTL7A |
| ADGRG5 | ACTN1 |
| ADORA3 | ACTN2 |
| ADPRHL1 | ACTN3 |
| ADTRP | ACVR1 |
| AFF2 | ACVR2A |
| AGBL2 | ACVRL1 |
| AGBL5 | ADAD2 |
| AGER | ADAM11 |
| AGMO | ADAM12 |
| AGPAT2 | ADAM19 |
| AGR2 | ADAM33 |
| AGR3 | ADAMTS1 |
| AGRN | ADAMTS16 |
| AGTRAP | ADAMTS19 |
| AHCY | ADAMTS2 |
| AIFM1 | ADAMTS4 |
| AK2 | ADAMTS5 |
| AK7 | ADAMTS8 |
| AK8 | ADAMTS9 |
| ALAD | ADAMTSL1 |
| ALDH18A1 | ADAMTSL4 |
| ALDH3A1 | ADAMTSL5 |
| ALDH3B2 | ADARB1 |
| ALDH5A1 | ADCY3 |
| ALDOA | ADCY4 |
| ALG10 | ADCY5 |
| ALG3 | ADD1 |
| ALG8 | ADGRA2 |
| ALKBH4 | ADGRE5 |
| ALOX15B | ADGRF5 |
| ALS2CL | ADGRG4 |
| ALX3 | ADGRL4 |
| AMBP | ADH1A |
| AMDHD1 | ADH1B |
| AMER3 | ADH1C |
| AMN | ADH5 |
| AMPD3 | ADIPOQ |
| AMY1C | ADIRF |
| AMY2A | ADPRH |
| AMY2B | ADPRM |
| ANGPTL6 | ADRA2A |
| ANK3 | ADRA2B |
| ANKEF1 | ADRB2 |
| ANKRD16 | AEBP1 |
| ANKRD18A | AES |
| ANKRD20A2 | AFAP1L2 |
| ANKRD22 | AGO4 |
| ANKRD23 | AGPAT4 |
| ANKRD65 | AGPAT4-IT1 |
| ANKRD7 | AGT |
| ANKZF1 | AGTR1 |
| ANLN | AGTR2 |
| ANOS1 | AHNAK |
| AOC1 | AHSP |
| AP1G2 | AK6 |
| AP1M2 | AKAP12 |
| AP1S3 | AKAP13 |
| AP3M2 | AKR1C3 |
| AP5B1 | AKT3 |
| APEH | AKTIP |
| APITD1 | ALAS2 |
| APOBEC3B | ALDH1A1 |
| APOBEC3D | ALDH1A3 |
| APOC1 | ALDH1B1 |
| APOC2 | ALDH2 |
| APOL1 | ALDH3A2 |
| APOL4 | ALOX5AP |
| APOO | AMFR |
| APOOL | AMOTL1 |
| APRT | AMOTL2 |
| AQP11 | AMPH |
| AQP5 | ANG |
| ARHGAP11A | ANGPT1 |
| ARHGAP8 | ANGPT2 |
| ARHGEF16 | ANGPTL1 |
| ARHGEF19 | ANGPTL2 |
| ARHGEF39 | ANGPTL4 |
| ARHGEF5 | ANK2 |
| ARID2 | ANKRD13B |
| ARL14 | ANKRD29 |
| ARMC10 | ANKRD35 |
| ARMC3 | ANKRD50 |
| ARMC7 | ANKRD53 |
| ARNTL2 | ANKS1B |
| ARPC1B | ANO1 |
| ARRDC1 | ANTXR1 |
| ARSD | ANTXR2 |
| ARSE | ANXA5 |
| ASAP2 | ANXA6 |
| ASB15 | AOC3 |
| ASB6 | AOX1 |
| ASB7 | AP1S2 |
| ASF1B | AP4S1 |
| ASIC2 | APBB1 |
| ASPHD1 | APC |
| ASPHD2 | APC2 |
| ASPM | APOD |
| ASRGL1 | APOL3 |
| ASS1 | APOLD1 |
| ATAD2 | APP |
| ATAD5 | APPBP2 |
| ATF7IP | AQP1 |
| ATF7IP2 | AQP9 |
| ATG4D | ARAP3 |
| ATIC | ARC |
| ATL3 | ARHGAP1 |
| ATOH1 | ARHGAP10 |
| ATP13A1 | ARHGAP15 |
| ATP1B1 | ARHGAP17 |
| ATP23 | ARHGAP21 |
| ATP2B3 | ARHGAP22 |
| ATP2C2 | ARHGAP24 |
| ATP4A | ARHGAP44 |
| ATP5J2 | ARHGAP9 |
| ATP6AP1 | ARHGDIB |
| ATP6V0A4 | ARHGEF10 |
| ATP6V1B1 | ARHGEF15 |
| ATP6V1C2 | ARHGEF17 |
| ATP7B | ARHGEF25 |
| ATP9A | ARHGEF26 |
| ATR | ARHGEF4 |
| ATXN2L | ARHGEF6 |
| AUNIP | ARID5B |
| AURKA | ARL13B |
| AURKB | ARL15 |
| AVL9 | ARL2BP |
| AXDND1 | ARL6IP5 |
| B3GNT3 | ARMC9 |
| B4GALNT4 | ARMCX1 |
| BAG4 | ARMCX3 |
| BAIAP2 | ARNT |
| BAIAP2L1 | ARNTL |
| BAIAP3 | ARR3 |
| BAK1 | ARSB |
| BARX2 | ARSI |
| BATF2 | ART4 |
| BBOX1 | ART5 |
| BCAS4 | ASB2 |
| BCL11A | ASGR1 |
| BCL2L1 | ASIC1 |
| BCL2L11 | ASMT |
| BCL2L14 | ASMTL |
| BCO1 | ASPA |
| BDH1 | ASPN |
| BHLHA15 | ASPRV1 |
| BICD1 | ATF4 |
| BIK | ATG10 |
| BIRC5 | ATL1 |
| BIRC8 | ATOH8 |
| BLM | ATP10D |
| BLNK | ATP11B |
| BMF | ATP1A2 |
| BMP7 | ATP1B3 |
| BMS1P5 | ATP6V1B2 |
| BOP1 | ATP6V1G2 |
| BORA | ATP8B2 |
| BPNT1 | ATP8B4 |
| BRAF | AVEN |
| BRCA2 | AVPR1A |
| BRCC3 | AXIN2 |
| BRI3BP | AXL |
| BROX | AZI2 |
| BSCL2 | AZIN2 |
| BSPRY | B3GNT5 |
| BTBD3 | B3GNT9 |
| BTG4 | BACE1 |
| BUB1 | BACH1 |
| BUB1B | BACH2 |
| BZW2 | BAG2 |
| C10orf35 | BAG3 |
| C10orf95 | BAHCC1 |
| C10orf99 | BAMBI |
| C11orf16 | BARHL2 |
| C11orf45 | BASP1 |
| C11orf52 | BATF3 |
| C11orf54 | BBS9 |
| C11orf65 | BCHE |
| C11orf80 | BCL11B |
| C12orf60 | BCL2 |
| C12orf65 | BCL7B |
| C12orf66 | BCR |
| C14orf105 | BDH2 |
| C14orf79 | BDKRB1 |
| C14orf93 | BDKRB2 |
| C15orf48 | BDNF |
| C16orf59 | BEGAIN |
| C17orf53 | BEND6 |
| C18orf25 | BEST1 |
| C19orf33 | BEST3 |
| C19orf48 | BGN |
| C19orf57 | BHMT2 |
| C1GALT1 | BIN3-IT1 |
| C1orf106 | BMI1 |
| C1orf112 | BMP15 |
| C1orf116 | BMP2 |
| C1orf186 | BMP4 |
| C1orf226 | BMP6 |
| C1orf35 | BMP8A |
| C1orf74 | BMX |
| C1QL2 | BNC2 |
| C1RL | BNIP3L |
| C20orf141 | BOC |
| C20orf96 | BOK |
| C21orf58 | BORCS7 |
| C22orf24 | BPIFA2 |
| C22orf29 | BTD |
| C2orf15 | BTG1 |
| C2orf47 | BTG2 |
| C2orf54 | BVES |
| C2orf88 | C10orf54 |
| C4BPA | C11orf24 |
| C4orf19 | C11orf63 |
| C5orf30 | C12orf54 |
| C6orf136 | C12orf57 |
| C6orf15 | C14orf132 |
| C6orf223 | C15orf41 |
| C7orf49 | C16orf45 |
| C8G | C16orf89 |
| C8orf31 | C1orf123 |
| C9orf116 | C1orf198 |
| C9orf16 | C1orf21 |
| C9orf3 | C1orf216 |
| C9orf43 | C1orf54 |
| C9orf66 | C1QL4 |
| CA12 | C1QTNF1 |
| CA13 | C1QTNF2 |
| CA14 | C1QTNF3 |
| CA2 | C1QTNF4 |
| CA8 | C1QTNF5 |
| CA9 | C1QTNF5///MFRP |
| CABYR | C1QTNF7 |
| CACNA2D3 | C1R |
| CACNB4 | C1S |
| CADM1 | C21orf2 |
| CADM4 | C22orf31 |
| CALCB | C2CD2 |
| CALML6 | C2orf40 |
| CALR | C3orf18 |
| CAMK2B | C3orf36 |
| CAMSAP1 | C4orf32 |
| CAPG | C5AR1 |
| CAPN1 | C6 |
| CAPN13 | C7 |
| CAPN8 | C8orf46 |
| CAPN9 | C8orf48 |
| CAPS | C8orf58 |
| CAPSL | C9orf40 |
| CARD11 | C9orf72 |
| CARD14 | CA3 |
| CARD9 | CAB39L |
| CARMIL1 | CACNA1C |
| CASC1 | CACNA1G |
| CASP6 | CACNA1H |
| CATSPER3 | CACNB1 |
| CATSPERB | CACNB2 |
| CATSPERD | CALCOCO2 |
| CBFA2T2 | CALD1 |
| CBLC | CALHM2 |
| CBS | CAMK1 |
| CBX8 | CAMK2N1 |
| CCDC113 | CAP2 |
| CCDC120 | CAPN2 |
| CCDC125 | CARD8 |
| CCDC138 | CASQ2 |
| CCDC140 | CAST |
| CCDC142 | CAV1 |
| CCDC150 | CAV2 |
| CCDC18 | CAV3 |
| CCDC24 | CBFA2T3 |
| CCDC40 | CBLN4 |
| CCDC51 | CBR3 |
| CCDC57 | CBX7 |
| CCDC77 | CCDC102B |
| CCHCR1 | CCDC107 |
| CCL20 | CCDC116 |
| CCL25 | CCDC136 |
| CCL28 | CCDC149 |
| CCNA2 | CCDC3 |
| CCNB1 | CCDC69 |
| CCNB2 | CCDC80 |
| CCNB3 | CCDC81 |
| CCNE1 | CCDC82 |
| CCNF | CCDC88A |
| CCNO | CCDC92 |
| CCP110 | CCIN |
| CCSAP | CCL1 |
| CCT5 | CCL11 |
| CD177 | CCL19 |
| CD24 | CCL2 |
| CD2AP | CCL21 |
| CD38 | CCL23 |
| CD47 | CCL26 |
| CD72 | CCL8 |
| CD80 | CCM2L |
| CD82 | CCND2 |
| CDC20 | CCPG1 |
| CDC25C | CCR10 |
| CDC45 | CCRL2 |
| CDC6 | CCSER2 |
| CDC7 | CD163L1 |
| CDCA2 | CD1C |
| CDCA3 | CD1D |
| CDCA4 | CD200 |
| CDCA5 | CD248 |
| CDCA7 | CD28 |
| CDCA8 | CD302 |
| CDCP1 | CD34 |
| CDH1 | CD36 |
| CDH16 | CD44 |
| CDH18 | CD59 |
| CDH24 | CD69 |
| CDH7 | CD83 |
| CDHR1 | CD93 |
| CDK1 | CD99 |
| CDK10 | CD99L2 |
| CDK18 | CDC42EP1 |
| CDK19 | CDC42EP2 |
| CDK20 | CDC42EP3 |
| CDK4 | CDH11 |
| CDK5 | CDH13 |
| CDKN2A | CDH17 |
| CDKN2B | CDH19 |
| CDKN3 | CDH23 |
| CDS1 | CDH5 |
| CDT1 | CDIP1 |
| CDX2 | CDK14 |
| CEACAM1 | CDK17 |
| CEACAM6 | CDKL1 |
| CECR6 | CDKN1A |
| CELF4 | CDO1 |
| CELSR1 | CDR2 |
| CELSR2 | CEACAM19 |
| CENPA | CEBPB |
| CENPBD1 | CEBPD |
| CENPE | CEBPE |
| CENPF | CELF2 |
| CENPH | CEP120 |
| CENPI | CEP170 |
| CENPK | CES1 |
| CENPL | CFD |
| CENPM | CFH |
| CENPN | CFHR1 |
| CENPO | CFHR2 |
| CENPU | CFHR3 |
| CEP250 | CFL2 |
| CEP44 | CFP |
| CEP55 | CGNL1 |
| CEP72 | CGREF1 |
| CES3 | CGRRF1 |
| CES5A | CH25H |
| CFAP43 | CHD1 |
| CFAP45 | CHD5 |
| CFAP47 | CHN1 |
| CFAP57 | CHPT1 |
| CFAP70 | CHRD |
| CFB | CHRDL1 |
| CFTR | CHRDL2 |
| CGN | CHRM3 |
| CHAF1A | CHRM4 |
| CHAF1B | CHRNA2 |
| CHD6 | CHST7 |
| CHDH | CHSY1 |
| CHEK1 | CHSY3 |
| CHI3L1 | CILP |
| CHI3L2 | CIRBP |
| CHKA | CKB |
| CHML | CLDN18 |
| CHMP4C | CLDN5 |
| CHODL | CLDND1 |
| CHRM5 | CLDND2 |
| CHRNA5 | CLEC10A |
| CHST4 | CLEC14A |
| CHST5 | CLEC1A |
| CHST6 | CLEC2B |
| CHST9 | CLEC3B |
| CHTF18 | CLEC4A |
| CIB1 | CLEC4D |
| CIITA | CLEC4G |
| CITED4 | CLIC2 |
| CKAP2 | CLIC4 |
| CKAP2L | CLIP1 |
| CKMT1B | CLIP3 |
| CKS2 | CLMP |
| CLCN2 | CLN8 |
| CLCN7 | CLSTN2 |
| CLDN10 | CMAHP |
| CLDN23 | CMTM2 |
| CLDN3 | CMTM3 |
| CLDN4 | CMTM5 |
| CLDN6 | CMYA5 |
| CLDN7 | CNGA3 |
| CLEC18B | CNN1 |
| CLEC19A | CNN3 |
| CLGN | CNOT9 |
| CLIC3 | CNPY4 |
| CLIC6 | CNR1 |
| CLINT1 | CNRIP1 |
| CLMN | CNTN1 |
| CLN3 | CNTN2 |
| CLN6 | CNTNAP1 |
| CLSPN | CNTNAP3 |
| CLUHP3 | COBLL1 |
| CLUL1 | COL12A1 |
| CMTM7 | COL14A1 |
| CMTM8 | COL15A1 |
| CNDP1 | COL16A1 |
| CNDP2 | COL1A1 |
| CNKSR1 | COL1A2 |
| CNNM4 | COL21A1 |
| CNPY2 | COL3A1 |
| COA4 | COL4A1 |
| COA6 | COL4A2 |
| COASY | COL4A5 |
| COBL | COL4A6 |
| COL23A1 | COL5A1 |
| COL24A1 | COL5A2 |
| COL9A2 | COL5A3 |
| COLEC11 | COL6A1 |
| COMMD9 | COL6A2 |
| COMTD1 | COL6A3 |
| CORO2A | COLGALT2 |
| CORT | COPA |
| COX15 | COPS4 |
| COX5A | COPZ2 |
| COX5B | COQ10B |
| COX6B1 | CORIN |
| COX7B | CORO2B |
| CP | COX4I2 |
| CPAMD8 | COX7A1 |
| CPLX1 | CPA3 |
| CPM | CPA5 |
| CPNE3 | CPA6 |
| CPNE7 | CPE |
| CPS1 | CPEB1 |
| CPSF3 | CPEB4 |
| CPT2 | CPED1 |
| CR1 | CPNE5 |
| CRABP1 | CPQ |
| CRB3 | CPT1C |
| CREB3L4 | CPVL |
| CRYBB3 | CPXM2 |
| CSE1L | CPZ |
| CSHL1 | CRADD |
| CSNK2A1 | CREB5 |
| CSNK2B | CREM |
| CST6 | CRHBP |
| CSTB | CRIM1 |
| CSTF1 | CRISPLD1 |
| CSTF2 | CRISPLD2 |
| CSTL1 | CRLF1 |
| CT45A1 | CRMP1 |
| CTAGE15 | CROT |
| CTAGE5 | CRTAP |
| CTNNA2 | CRTC3 |
| CTNND1 | CRY1 |
| CTPS2 | CRY2 |
| CTSA | CRYAB |
| CTSE | CRYBG3 |
| CTSV | CSF3 |
| CXADR | CSGALNACT1 |
| CXCL10 | CSGALNACT2 |
| CXCL11 | CSNK1A1 |
| CXCL13 | CSPG4 |
| CXCL16 | CSRNP1 |
| CXCL17 | CSRP1 |
| CXorf38 | CSRP2 |
| CXXC5 | CTGF |
| CYB561 | CTHRC1 |
| CYB561D2 | CTIF |
| CYBA | CTSF |
| CYC1 | CTSG |
| CYP2C18 | CTSK |
| CYP2C9 | CUTC |
| CYP2D6 | CXCL1 |
| CYP2J2 | CXCL12 |
| CYP2S1 | CXCL2 |
| CYP3A5 | CXCL8 |
| CYP3A7 | CXCR1 |
| CYP4B1 | CXCR2 |
| CYP4Z1 | CXorf36 |
| DAND5 | CXorf57 |
| DAPL1 | CYB5D1 |
| DARS2 | CYB5R3 |
| DBNDD1 | CYBRD1 |
| DBP | CYGB |
| DBT | CYP11B2 |
| DCAF11 | CYP1B1 |
| DCAF15 | CYP27A1 |
| DCAF17 | CYP2U1 |
| DCDC2 | CYP39A1 |
| DCLRE1A | CYP7B1 |
| DCLRE1C | CYR61 |
| DCXR | CYTH1 |
| DDIAS | CYTH4 |
| DDR1 | CYTL1 |
| DDX11 | CYYR1 |
| DDX11L5 | DAAM1 |
| DDX4 | DAB2 |
| DDX49 | DACH1 |
| DDX58 | DACH2 |
| DECR2 | DACT1 |
| DEFA4 | DACT3 |
| DEFB1 | DAPK3 |
| DEFB103B | DAZL |
| DENND1A | DCHS1 |
| DENND2D | DCLK1 |
| DEPDC1 | DCLK2 |
| DEPDC1B | DCN |
| DEPDC4 | DCTN6 |
| DEPTOR | DCUN1D3 |
| DERL1 | DDR2 |
| DERL3 | DDX3X |
| DGCR5 | DDX43 |
| DHCR24 | DDX5 |
| DHCR7 | DEFA3 |
| DHFR | DEFA6 |
| DHFR2 | DENND2A |
| DHRS13 | DENND2C |
| DHRS2 | DENND5A |
| DHTKD1 | DES |
| DHX33 | DFNA5 |
| DIAPH1 | DGKB |
| DIAPH3 | DGKD |
| DIP2B | DHRS7 |
| DIRC2 | DIAPH2 |
| DLG3 | DIO2 |
| DLGAP2 | DIO3 |
| DLGAP5 | DIO3OS |
| DLK1 | DIRAS1 |
| DLX4 | DIS3 |
| DLX6 | DISP1 |
| DMBX1 | DKK1 |
| DMKN | DKK2 |
| DMTN | DKK3 |
| DNAAF3 | DLC1 |
| DNAAF5 | DLG4 |
| DNAH6 | DLL1 |
| DNAH7 | DLL4 |
| DNAJA3 | DMD |
| DNAJB13 | DMPK |
| DNAJC14 | DMRT2 |
| DNAJC22 | DMRTC2 |
| DNAJC28 | DNAJB1 |
| DNAJC30 | DNAJB4 |
| DNAJC5 | DNAJB5 |
| DNASE1 | DNAJB9 |
| DNASE1L2 | DNAJC18 |
| DNMT1 | DNAJC27 |
| DNMT3A | DNASE1L3 |
| DNMT3B | DOCK1 |
| DOC2A | DOCK10 |
| DPCD | DOCK11 |
| DPF3 | DOCK4 |
| DPP3 | DOCK6 |
| DPPA2 | DOK6 |
| DPY30 | DOLPP1 |
| DPYSL5 | DPEP2 |
| DRC1 | DPP6 |
| DSCC1 | DPT |
| DSG2 | DPY19L2 |
| DSN1 | DPYD |
| DSP | DPYSL2 |
| DTL | DPYSL3 |
| DTNB | DPYSL4 |
| DTX2 | DSTN |
| DTX3L | DTNA |
| DUS1L | DTWD1 |
| DUS4L | DTX3 |
| DYDC2 | DUSP1 |
| DYX1C1 | DUSP22 |
| E2F1 | DUSP3 |
| E2F3 | DUSP5 |
| E2F5 | DUSP8 |
| E2F8 | DYNC1I1 |
| EAF1 | DYNC2H1 |
| EARS2 | DYRK3 |
| EBP | DYSF |
| ECE2 | DZIP1 |
| ECHDC3 | DZIP1L |
| ECI1 | EBF1 |
| ECT2 | ECM1 |
| EDARADD | ECM2 |
| EDEM1 | ECSCR |
| EDEM2 | EDIL3 |
| EDN2 | EDNRA |
| EDN3 | EDNRB |
| EEF1A2 | EDRF1 |
| EEF1E1 | EEA1 |
| EFCAB11 | EEPD1 |
| EFCAB3 | EFCC1 |
| EFCAB6 | EFEMP2 |
| EFHC2 | EFHD1 |
| EFNA1 | EFHD2 |
| EFNA4 | EFNB2 |
| EGFL6 | EFNB3 |
| EGLN3 | EFS |
| EHF | EGFL7 |
| EIF2AK1 | EGFLAM |
| EIF2AK2 | EGR1 |
| EIF4EBP1 | EGR2 |
| EIF5B | EGR3 |
| ELF3 | EHBP1 |
| ELK4 | EHBP1L1 |
| ELL3 | EHD1 |
| ELMO3 | EHD2 |
| ELMOD2 | EID1 |
| ELMSAN1 | EID3 |
| ELOVL3 | EIF1 |
| ELOVL6 | EIF1B |
| EME1 | ELK3 |
| EML2 | ELL2 |
| EML4 | ELN |
| ENC1 | EMCN |
| ENKUR | EMILIN1 |
| ENO1 | EMILIN2 |
| ENO3 | EML1 |
| ENOSF1 | EMP1 |
| ENPP5 | EMP3 |
| ENTPD2 | ENAH |
| ENTPD5 | ENAM |
| ENTPD6 | ENG |
| EPB41 | ENOX1 |
| EPB41L4B | ENPEP |
| EPB41L5 | ENPP1 |
| EPCAM | ENPP2 |
| EPHA1 | ENPP3 |
| EPN3 | EPAS1 |
| EPOR | EPB41L3 |
| EPPK1 | EPDR1 |
| EPS8L1 | EPHA3 |
| EPS8L2 | EPHA4 |
| EPSTI1 | EPHX3 |
| EPT1 | EPM2A |
| EPYC | EPS15 |
| ERBB2 | EPS8 |
| ERBB3 | ERG |
| ERI2 | ERO1B |
| ERICH5 | ERRFI1 |
| ERLIN2 | ESAM |
| ERMN | ESD |
| ERMP1 | ETS1 |
| ERO1A | ETS2 |
| ERP27 | EVA1B |
| ERVH-3 | EVI2B |
| ERVH-4 | EVL |
| ERVH48-1 | EXTL1 |
| ERVMER34-1 | EXTL2 |
| ESM1 | F10 |
| ESPL1 | F13A1 |
| ESPN | F2R |
| ESR2 | FAM107A |
| ESRP1 | FAM110B |
| ESRP2 | FAM110D |
| ESRRA | FAM114A1 |
| ESRRG | FAM114A2 |
| ETNK1 | FAM124A |
| ETV4 | FAM124B |
| ETV5 | FAM126A |
| EVA1A | FAM129A |
| EVPL | FAM13A |
| EXD3 | FAM13B |
| EXO1 | FAM162B |
| EXOC3L4 | FAM167B |
| EXOSC4 | FAM175A |
| EXOSC5 | FAM177A1 |
| EXPH5 | FAM184A |
| EZH2 | FAM189A2 |
| EZR | FAM198A |
| F12 | FAM198B |
| F8A1 | FAM19A5 |
| FA2H | FAM208A |
| FAAH | FAM212B |
| FAAH2 | FAM216A |
| FAAP24 | FAM26D |
| FABP6 | FAM26E |
| FABP7 | FAM43A |
| FADD | FAM46A |
| FAF2 | FAM46B |
| FAM110A | FAM49A |
| FAM120C | FAM50B |
| FAM122B | FAM65A |
| FAM134A | FAM65B |
| FAM136A | FAM71A |
| FAM13C | FAM76A |
| FAM173A | FAP |
| FAM174B | FAS |
| FAM181A | FAT1 |
| FAM187A | FAT4 |
| FAM199X | FAXDC2 |
| FAM200A | FBLIM1 |
| FAM213B | FBLN1 |
| FAM218A | FBLN2 |
| FAM227B | FBLN5 |
| FAM24B | FBLN7 |
| FAM3B | FBN1 |
| FAM3D | FBXL3 |
| FAM49B | FBXL7 |
| FAM57B | FBXO3 |
| FAM60A | FBXO30 |
| FAM64A | FCAR |
| FAM83A | FCER1A |
| FAM83D | FCGRT |
| FAM83E | FCN1 |
| FAM83F | FEM1B |
| FAM84B | FEM1C |
| FAM90A1 | FER |
| FAM91A1 | FERMT2 |
| FAM95C | FEZ1 |
| FAM96A | FEZ2 |
| FANCA | FFAR2 |
| FANCB | FGF1 |
| FANCC | FGF13 |
| FANCD2 | FGF2 |
| FANCF | FGF7 |
| FANCG | FGFBP2 |
| FANK1 | FGFR1 |
| FARP1 | FGL2 |
| FARSA | FGR |
| FASN | FHL1 |
| FASTK | FHL2 |
| FASTKD1 | FHL3 |
| FBP1 | FHL5 |
| FBXL16 | FHOD3 |
| FBXL18 | FIBIN |
| FBXL6 | FIGF |
| FBXO15 | FILIP1L |
| FBXO16 | FKBP14 |
| FBXO2 | FKBP9 |
| FBXO21 | FLI1 |
| FBXO27 | FLJ31713 |
| FBXO46 | FLNA |
| FBXO6 | FLNC |
| FBXW9 | FLRT2 |
| FCGBP | FLT3LG |
| FEN1 | FLT4 |
| FEV | FMO1 |
| FGA | FMO2 |
| FGF12 | FMO3 |
| FGF17 | FMO5 |
| FGF19 | FMOD |
| FGFBP1 | FN1 |
| FGFR1OP | FNDC4 |
| FGFR3 | FNDC5 |
| FHIT | FOLR2 |
| FKBP4 | FOS |
| FKBP5 | FOSB |
| FKBPL | FOSL1 |
| FLAD1 | FOSL2 |
| FLJ21369 | FOXF1 |
| FLJ23867 | FOXN3 |
| FLRT3 | FOXP1 |
| FLVCR1 | FPR1 |
| FLVCR2 | FREM1 |
| FMR1 | FRMD3 |
| FNBP1L | FRMD6 |
| FOLH1 | FRY |
| FOLR1 | FRZB |
| FOLR3 | FST |
| FOXA1 | FSTL1 |
| FOXA2 | FSTL3 |
| FOXA3 | FUNDC2 |
| FOXJ1 | FXYD1 |
| FOXM1 | FXYD5 |
| FRAS1 | FXYD6 |
| FRAT2 | FYCO1 |
| FRK | FYN |
| FRMD5 | FZD4 |
| FSBP | FZD7 |
| FSD1L | FZD8 |
| FUK | G0S2 |
| FUT2 | GAB1 |
| FUT3 | GAB3 |
| FUT5 | GABARAPL1 |
| FUT9 | GABBR1 |
| FUZ | GABRA4 |
| FXYD3 | GADD45A |
| FXYD4 | GADD45B |
| FZD10 | GADD45G |
| FZD3 | GALNT13 |
| FZD5 | GALNT15 |
| G6PC | GALNT16 |
| GAD1 | GALNT18 |
| GAL | GARNL3 |
| GAL3ST1 | GAS1 |
| GALE | GAS6 |
| GALM | GAS7 |
| GALNT12 | GATA2 |
| GALNT14 | GATA6 |
| GALNT2 | GATS |
| GALNT4 | GCKR |
| GALNT6 | GDF10 |
| GALP | GDF6 |
| GAPDH | GDPD5 |
| GART | GEM |
| GAS8 | GFAP |
| GATAD2B | GFOD1 |
| GATC | GFPT2 |
| GBP3 | GFRA1 |
| GCAT | GGT5 |
| GCG | GGTA1P |
| GCHFR | GHR |
| GCNT1 | GIMAP1 |
| GCNT3 | GIMAP4 |
| GCSH | GIMAP5 |
| GDA | GIMAP6 |
| GDF11 | GIMAP8 |
| GDPD2 | GIPC2 |
| GEMIN6 | GJA1 |
| GEMIN7 | GJA4 |
| GET4 | GJA5 |
| GFER | GJA9 |
| GFRA4 | GJB3 |
| GGCT | GJC1 |
| GIGYF1 | GJC2 |
| GINS2 | GLI1 |
| GINS4 | GLI2 |
| GIPC1 | GLI3 |
| GJB1 | GLIPR1 |
| GJB2 | GLIPR2 |
| GLCCI1 | GLIS1 |
| GLDC | GLS |
| GLE1 | GLT1D1 |
| GLI4 | GLT8D2 |
| GLMP | GMFG |
| GLS2 | GNA14 |
| GLYATL1 | GNAI1 |
| GMCL1 | GNAZ |
| GMDS | GNB3 |
| GMNN | GNB4 |
| GMPR | GNG11 |
| GMPS | GNG12 |
| GNL3L | GNG2 |
| GOLGA8B | GNG7 |
| GOLT1A | GNPDA2 |
| GON7 | GOLGA4 |
| GOSR1 | GOLIM4 |
| GOT1 | GP1BA |
| GP6 | GP1BB |
| GPAA1 | GP5 |
| GPD2 | GPAT3 |
| GPI | GPER1 |
| GPR107 | GPIHBP1 |
| GPR143 | GPM6A |
| GPR160 | GPNMB |
| GPR180 | GPR132 |
| GPR19 | GPR137B |
| GPR27 | GPR146 |
| GPR35 | GPR161 |
| GPR78 | GPR176 |
| GPR87 | GPR183 |
| GPRC5B | GPR21 |
| GPRC5C | GPR34 |
| GPRIN2 | GPR4 |
| GPT | GPR65 |
| GPT2 | GPRASP1 |
| GPX1 | GPRASP2 |
| GRAMD1C | GPSM1 |
| GRB14 | GPX8 |
| GRB7 | GRAP |
| GRHL1 | GRASP |
| GRHL2 | GRB10 |
| GRHL3 | GREB1 |
| GRIN3B | GREM1 |
| GRM8 | GREM2 |
| GRTP1 | GRID1 |
| GSDMB | GRIN2C |
| GSDMC | GRK5 |
| GSG1L | GSN |
| GSR | GSTA4 |
| GSS | GSTM5 |
| GSTA1 | GSTT2 |
| GSTA3 | GTF2IRD2 |
| GSTA5 | GUCA1A |
| GSTK1 | GUCY1A2 |
| GSTO2 | GUCY1A3 |
| GSTP1 | GULP1 |
| GTF2H3 | GYG1 |
| GTF2H4 | GYPC |
| GTF2IRD1 | GZF1 |
| GTF3C2 | GZMK |
| GTF3C4 | HAAO |
| GTPBP3 | HABP4 |
| GTSE1 | HACD1 |
| GTSF1 | HAND2 |
| GUCA2B | HAND2-AS1 |
| GUF1 | HAS1 |
| H2AFX | HAS2 |
| H2AFY | HBA1 |
| HACD2 | HBA2 |
| HACD3 | HBB |
| HAGHL | HBD |
| HARBI1 | HBE1 |
| HAUS6 | HBEGF |
| HCP5 | HBG1 |
| HDDC3 | HBM |
| HDGF | HBQ1 |
| HDHD3 | HCFC2 |
| HELLS | HCK |
| HENMT1 | HCN4 |
| HES2 | HDAC4 |
| HESX1 | HDC |
| HEXIM2 | HDGFRP3 |
| HGD | HECA |
| HGH1 | HECTD2 |
| HILPDA | HEG1 |
| HINT2 | HEPH |
| HIPK2 | HERC1 |
| HIST1H1C | HERC3 |
| HIST1H1D | HES4 |
| HIST1H1E | HEYL |
| HIST1H2AB | HGF |
| HIST1H2AD | HHEX |
| HIST1H2AE | HIC1 |
| HIST1H2AG | HIGD1B |
| HIST1H2AH | HIST1H4G |
| HIST1H2AI | HLF |
| HIST1H2AK | HLX |
| HIST1H2AL | HMCN1 |
| HIST1H2BB | HMCN2 |
| HIST1H2BC | HMGN5 |
| HIST1H2BD | HOPX |
| HIST1H2BE | HOXA10 |
| HIST1H2BF | HOXA13 |
| HIST1H2BG | HOXC10 |
| HIST1H2BH | HOXC9 |
| HIST1H2BI | HOXD10 |
| HIST1H2BJ | HOXD11 |
| HIST1H2BK | HPGD |
| HIST1H2BL | HPGDS |
| HIST1H2BM | HRC |
| HIST1H2BN | HRCT1 |
| HIST1H2BO | HRH4 |
| HIST1H3B | HS6ST3 |
| HIST1H3D | HSD11B1 |
| HIST1H3E | HSD17B11 |
| HIST1H3F | HSD17B14 |
| HIST1H3H | HSD17B6 |
| HIST1H3I | HSPA12B |
| HIST1H4C | HSPA13 |
| HIST1H4F | HSPA1A |
| HIST1H4H | HSPA1L |
| HIST1H4I | HSPA2 |
| HIST1H4J | HSPA6 |
| HIST1H4K | HSPB2 |
| HIST1H4L | HSPB3 |
| HIST2H2AA3 | HSPB7 |
| HIST2H2AB | HSPB8 |
| HIST2H2AC | HSPG2 |
| HIST2H2BE | HTN3 |
| HIST2H3A///HIST2H3C | HTR2A |
| HIST2H4A | HTR2B |
| HIST2H4B///HIST2H4A | HTR2C |
| HIST3H2A | HTRA1 |
| HIST3H2BB | HTRA3 |
| HJURP | HVCN1 |
| HK2 | HYAL1 |
| HKDC1 | ICA1L |
| HLA-DMB | ICAM1 |
| HLA-DOA | ICAM2 |
| HLA-DOB | ICAM4 |
| HLA-DPB2 | ICOS |
| HMBS | ID1 |
| HMGA2 | ID2 |
| HMGB2 | ID3 |
| HMGB3 | IDS |
| HMGB3P1 | IER2 |
| HMGCR | IER3 |
| HMMR | IER5 |
| HN1 | IFRD1 |
| HN1L | IGDCC4 |
| HOMER2 | IGF1 |
| HOMEZ | IGF2 |
| HOOK1 | IGFBP4 |
| HOOK2 | IGFBP5 |
| HOXA1 | IGFBP6 |
| HOXB5 | IGFBP7 |
| HOXB6 | IGSF10 |
| HOXB7 | IGSF21 |
| HOXB8 | IHH |
| HOXB9 | IKBIP |
| HOXC4 | IL10RA |
| HOXC8 | IL16 |
| HOXD1 | IL17B |
| HPDL | IL17D |
| HPN | IL17RD |
| HPSE | IL18RAP |
| HRASLS | IL1B |
| HRASLS2 | IL1F10 |
| HRK | IL1R1 |
| HSD17B1 | IL20 |
| HSD17B2 | IL3 |
| HSD17B8 | IL33 |
| HSH2D | IL34 |
| HSPB9 | IL3RA |
| HSPE1 | IL4R |
| HTATIP2 | IL6 |
| HTR1A | IL6ST |
| HTR1D | IL7R |
| HTR2C | ILK |
| HTR3A | INHBA |
| HYAL3 | INMT |
| HYDIN | INPP1 |
| IDH1 | INPP4B |
| IDH2 | INPP5A |
| IDO1 | INPP5D |
| IFI27 | INS |
| IFI30 | INSIG1 |
| IFITM1 | IPO5 |
| IFNE | IQCF5 |
| IFNL2 | IQGAP1 |
| IFNLR1 | IQSEC1 |
| IFT140 | IRAK3 |
| IFT22 | IRX5 |
| IGF2BP1 | ISCU |
| IGF2BP2 | ISLR |
| IGF2BP3 | ITGA1 |
| IGFALS | ITGA11 |
| IGFBP1 | ITGA5 |
| IGHA1 | ITGA6 |
| IGSF3 | ITGA7 |
| IGSF9 | ITGA8 |
| IKBKB | ITGA9 |
| IKBKE | ITGAM |
| IL12A | ITGAV |
| IL17RE | ITGB1 |
| IL18 | ITGB1BP1 |
| IL1RAP | ITGB1BP2 |
| IL20RB | ITGB5 |
| IL22RA1 | ITGBL1 |
| IL23A | ITIH3 |
| IL36A | ITIH5 |
| IL4I1 | ITM2A |
| IL9 | ITPKB |
| IMMP1L | ITPR1 |
| IMPA2 | ITPRIP |
| IMPG1 | ITSN1 |
| INA | JAM2 |
| INCENP | JAM3 |
| INHA | JAML |
| INPPL1 | JDP2 |
| IPMK | JOSD1 |
| IPO4 | JPH2 |
| IPP | JUN |
| IQCA1 | JUNB |
| IQCC | JUND |
| IQCD | KALRN |
| IQCF1 | KANK1 |
| IQCG | KANK2 |
| IQCK | KANK3 |
| IRAK2 | KATNAL1 |
| IRF3 | KATNBL1 |
| IRF5 | KBTBD11 |
| IRF6 | KBTBD8 |
| IRX3 | KCNAB1 |
| ISCA2 | KCNB1 |
| ISG15 | KCND1 |
| ISG20 | KCND2 |
| ISYNA1 | KCNE4 |
| ITCH | KCNJ8 |
| ITGA2B | KCNK6 |
| ITGA3 | KCNMB1 |
| ITGB3BP | KCNN1 |
| ITGB6 | KCNN2 |
| ITGB7 | KCNN3 |
| ITGB8 | KCTD10 |
| ITPKA | KCTD12 |
| ITPR3 | KCTD15 |
| JAK3 | KCTD17 |
| JMJD4 | KCTD20 |
| JUP | KCTD9 |
| KCNC1 | KDELC1 |
| KCNE3 | KDELC2 |
| KCNG2 | KHK |
| KCNJ16 | KIAA0226L |
| KCNJ5 | KIAA0355 |
| KCNJ6 | KIAA0408 |
| KCNK1 | KIAA0922 |
| KCNK13 | KIAA1324L |
| KCNK15 | KIAA1644 |
| KCNK16 | KIFAP3 |
| KCNK2 | KIRREL3 |
| KCNK5 | KLC1 |
| KCNMB2 | KLF10 |
| KCTD1 | KLF11 |
| KCTD14 | KLF12 |
| KDF1 | KLF2 |
| KDM1B | KLF4 |
| KDM4A | KLF6 |
| KDM4D | KLF7 |
| KEL | KLF9 |
| KHNYN | KLHDC1 |
| KIAA0101 | KLHL13 |
| KIAA0907 | KLHL21 |
| KIAA1468 | KLHL29 |
| KIAA1549 | KLHL4 |
| KIAA1551 | KLHL5 |
| KIF11 | KLRB1 |
| KIF13B | KLRG1 |
| KIF14 | KPNA3 |
| KIF15 | KRT1 |
| KIF16B | KRT222 |
| KIF18A | KRTAP9-8 |
| KIF1A | LAMA2 |
| KIF20A | LAMA4 |
| KIF20B | LAMB1 |
| KIF21A | LAMB2 |
| KIF22 | LARGE1 |
| KIF23 | LARP6 |
| KIF24 | LATS2 |
| KIF2C | LAYN |
| KIF4A | LBH |
| KIFC1 | LCAT |
| KIFC2 | LCP2 |
| KLF5 | LDB2 |
| KLHDC7B | LDB3 |
| KLHDC8A | LDLRAD4 |
| KLHDC9 | LEFTY2 |
| KLHL24 | LEP |
| KLHL35 | LFNG |
| KLK1 | LGALS1 |
| KLK10 | LGALS12 |
| KLK11 | LGALS2 |
| KLK2 | LGI2 |
| KLK5 | LGI4 |
| KLK6 | LHFP |
| KLK7 | LHFPL2 |
| KLK8 | LHX6 |
| KLRC3 | LILRA5 |
| KMT5C | LILRB2 |
| KNL1 | LILRB3 |
| KNSTRN | LIMA1 |
| KNTC1 | LIMS2 |
| KPNA2 | LIN7A |
| KREMEN2 | LIN7B |
| KRT12 | LINC00152 |
| KRT15 | LINC00260 |
| KRT16 | LINC00341 |
| KRT17 | LINC00597 |
| KRT18 | LINC00982 |
| KRT19 | LINGO2 |
| KRT23 | LIPA |
| KRT33A | LIPE |
| KRT5 | LIX1L |
| KRT6A | LMCD1 |
| KRT6C | LMNA |
| KRT7 | LMO2 |
| KRT8 | LMOD1 |
| KRTCAP3 | LOC100128482 |
| KRTDAP | LOC102724428 |
| L2HGDH | LOC338620 |
| L3MBTL1 | LONRF1 |
| LACE1 | LONRF3 |
| LACTB2 | LOXL1 |
| LAD1 | LOXL2 |
| LAMA5 | LOXL3 |
| LAMB3 | LOXL4 |
| LAMC2 | LPAR1 |
| LAMP3 | LPAR6 |
| LAPTM4B | LPL |
| LARGE2 | LPP |
| LARP4B | LRCH1 |
| LBHD1 | LRCH2 |
| LCA5L | LRFN5 |
| LCAL1 | LRMP |
| LCE2C | LRP1 |
| LCN12 | LRP12 |
| LCN2 | LRRC17 |
| LCT | LRRC2 |
| LDAH | LRRC32 |
| LDHC | LRRC49 |
| LDHD | LRRC70 |
| LEFTY1 | LRRC8A |
| LEXM | LRRC8C |
| LGALS14 | LRRFIP1 |
| LGALS7 | LRRK2 |
| LGR4 | LRRN3 |
| LGR5 | LRRN4CL |
| LIG1 | LSAMP |
| LIMK1 | LSP1 |
| LINC00115 | LSS |
| LINC00328 | LTBP1 |
| LINC00467 | LTBP2 |
| LINC00588 | LTBP4 |
| LINC00839 | LTC4S |
| LINC01089 | LUM |
| LINC01106 | LXN |
| LINC01138 | LY96 |
| LIPG | LYL1 |
| LIPH | LYPLAL1 |
| LLGL2 | LYSMD2 |
| LMAN1 | LYST |
| LMLN | LYVE1 |
| LMNB1 | LYZ |
| LMNB2 | LZTS1 |
| LMO4 | MAEL |
| LMO7 | MAF |
| LMTK2 | MAFF |
| LNPEP | MAFG |
| LNX1 | MAFK |
| LOC100132356 | MAGEH1 |
| LOC100506282 | MAGI2 |
| LOC105371374 | MAMDC2 |
| LOC171391 | MAN1C1 |
| LOC728743 | MAOB |
| LOC81691 | MAP1A |
| LONP2 | MAP1B |
| LPAR2 | MAP1LC3B |
| LPAR3 | MAP3K3 |
| LPCAT3 | MAP3K7CL |
| LRAT | MAP3K8 |
| LRBA | MAP6 |
| LRFN4 | MAP7D1 |
| LRG1 | MAP7D3 |
| LRGUK | MAPK10 |
| LRIF1 | MAPK11 |
| LRP8 | MAPK12 |
| LRRC1 | MAPK8IP3 |
| LRRC19 | MAPRE1 |
| LRRC43 | MAPRE2 |
| LRRC45 | MAPT |
| LRRC6 | MARCKS |
| LRRC61 | MARK1 |
| LRRC8D | MARVELD1 |
| LRRC8E | MASP1 |
| LRRIQ1 | MAST4 |
| LRRTM1 | MAT2A |
| LSM4 | MATN2 |
| LSR | MBNL1 |
| LY6D | MBNL2 |
| LY6E | MCAM |
| LY75 | MCC |
| LYG1 | MCEMP1 |
| LYG2 | MCF2L |
| LYPD1 | MCHR1 |
| LYPD3 | MCL1 |
| LYPD6 | MCTP1 |
| LYPD6B | MDGA1 |
| LYPLA2 | ME1 |
| LZTFL1 | ME3 |
| MACROD1 | MED13L |
| MAD2L1 | MEDAG |
| MADCAM1 | MEF2A |
| MAGEA3 | MEF2C |
| MAGEB2 | MEIS2 |
| MAGIX | MEOX1 |
| MAGT1 | MEOX2 |
| MAL | METRNL |
| MAL2 | MEX3B |
| MANEAL | MFAP2 |
| MAP10 | MFAP4 |
| MAP2K5 | MFNG |
| MAP2K6 | MGARP |
| MAP3K1 | MGC24103 |
| MAP3K13 | MGP |
| MAP6D1 | MICAL1 |
| MAP7 | MICAL2 |
| MAPK13 | MID1 |
| MAPK15 | MID2 |
| MAPKAPK3 | MIDN |
| MARCKSL1 | MLANA |
| MARK2 | MLKL |
| MARVELD3 | MLLT11 |
| MAST1 | MMGT1 |
| MASTL | MMP16 |
| MAT1A | MMP19 |
| MAZ | MMP2 |
| MB | MMP23B |
| MBD6 | MMP28 |
| MBTD1 | MMRN1 |
| MBTPS2 | MMRN2 |
| MC1R | MN1 |
| MC4R | MNDA |
| MCAT | MOB2 |
| MCCC1 | MORC1 |
| MCCC2 | MORC4 |
| MCF2 | MORF4L1 |
| MCM10 | MORF4L2 |
| MCM2 | MORN5 |
| MCM3 | MPDZ |
| MCM3AP-AS1 | MPP4 |
| MCM4 | MPRIP |
| MCM5 | MRAS |
| MCM6 | MRFAP1 |
| MCM7 | MRGPRF |
| MCM8 | MRGPRX4 |
| MCM9 | MRVI1 |
| MCOLN2 | MS4A2 |
| MCOLN3 | MS4A4A |
| MCRIP2 | MS4A6A |
| MDK | MS4A6E |
| MECOM | MS4A7 |
| MED13L | MSANTD3 |
| MEGF8 | MSC |
| MELK | MSN |
| MEN1 | MSRA |
| MESP1 | MSX1 |
| MEST | MTMR6 |
| METTL13 | MTMR9LP |
| METTL9 | MTRF1L |
| MFSD12 | MTURN |
| MFSD3 | MUM1L1 |
| MFSD4B | MXD1 |
| MFSD6L | MXRA8 |
| MGA | MYADM |
| MGAT4B | MYC |
| MGME1 | MYCT1 |
| MICB | MYH1 |
| MIEF2 | MYH11 |
| MIF | MYH2 |
| MIPEP | MYH3 |
| MIS18A | MYH9 |
| MISP | MYL2 |
| MKI67 | MYL4 |
| MKRN1 | MYL6 |
| MLEC | MYL9 |
| MLXIPL | MYLK |
| MMEL1 | MYO15B |
| MMP1 | MYO1B |
| MMP10 | MYO1C |
| MMP12 | MYO1F |
| MMP15 | MYOC |
| MMP7 | MYOCD |
| MND1 | MYOM1 |
| MNS1 | MYOM2 |
| MOCOS | MYOT |
| MOCS1 | MYRIP |
| MOGAT1 | MYZAP |
| MOGS | NAALAD2 |
| MORN3 | NAALADL1 |
| MOSPD3 | NACAD |
| MOV10 | NACC2 |
| MPHOSPH9 | NAIP |
| MPP7 | NAMPT |
| MPZL2 | NAP1L2 |
| MREG | NAP1L3 |
| MRM1 | NAP1L5 |
| MRPL12 | NAV1 |
| MRPL13 | NAV3 |
| MRPL15 | NBEA |
| MRPL17 | NBL1 |
| MRPL19 | NCAM1 |
| MRPL2 | NCF2 |
| MRPL23 | NCF4 |
| MRPL3 | NDEL1 |
| MRPL30 | NDN |
| MRPL57 | NDNF |
| MRPS12 | NDRG2 |
| MRPS18B | NEDD4 |
| MRPS25 | NEDD9 |
| MRPS26 | NEFM |
| MRPS33 | NEGR1 |
| MRPS34 | NEK1 |
| MRPS35 | NEK7 |
| MRS2 | NENF |
| MSH2 | NES |
| MSH5 | NEUROD1 |
| MSI2 | NEUROD4 |
| MSLN | NEXN |
| MST1R | NFATC1 |
| MT1F | NFATC4 |
| MT3 | NFIA |
| MTA3 | NFIB |
| MTAP | NFIC |
| MTBP | NFIL3 |
| MTERF1 | NFKB1 |
| MTF2 | NFKBIA |
| MTFR2 | NFKBID |
| MTG1 | NFKBIZ |
| MTHFSD | NGF |
| MTIF2 | NGFR |
| MTMR1 | NID1 |
| MUC1 | NID2 |
| MUC16 | NKAIN2 |
| MUC20 | NKAPL |
| MUC4 | NKD1 |
| MYB | NKIRAS1 |
| MYBL2 | NLGN2 |
| MYBPC2 | NLRP1 |
| MYBPH | NLRP3 |
| MYCBP | NMT2 |
| MYCN | NMUR1 |
| MYCNOS | NNAT |
| MYEF2 | NNMT |
| MYH6 | NOD1 |
| MYO10 | NOS3 |
| MYO15A | NOSTRIN |
| MYO19 | NOTCH4 |
| MYO6 | NOV |
| MZT2B | NOVA1 |
| NAA40 | NOVA2 |
| NADSYN1 | NOX4 |
| NAPG | NPAS4 |
| NAPSA | NPDC1 |
| NCAPD2 | NPFF |
| NCAPG | NPHS2 |
| NCAPG2 | NPNT |
| NCAPH | NPR2 |
| NCBP2 | NPTN |
| NCBP2-AS2 | NPY1R |
| NCOA2 | NPY4R |
| NCOA3 | NPY5R |
| NDC1 | NR2F1 |
| NDUFA7 | NR2F2 |
| NDUFS8 | NR3C1 |
| NDUFV1 | NR3C2 |
| NECAB3 | NR4A1 |
| NECTIN1 | NR4A2 |
| NECTIN4 | NRN1 |
| NEDD4L | NRP1 |
| NEIL3 | NRP2 |
| NEK10 | NSRP1 |
| NEK11 | NT5E |
| NEK2 | NTAN1 |
| NELFCD | NTF3 |
| NEURL3 | NTM |
| NEUROG3 | NTRK1 |
| NFAM1 | NTRK2 |
| NFATC2IP | NTRK3 |
| NFE2L3 | NUAK1 |
| NIF3L1 | NUDT10 |
| NIPSNAP1 | NUDT11 |
| NKAIN1 | NXF1 |
| NKX2-2 | NXPE3 |
| NKX6-2 | OAF |
| NLGN4Y | OAT |
| NLK | ODF3 |
| NLRP2 | OGN |
| NLRP5 | OLFM1 |
| NME6 | OLFML1 |
| NME9 | OLFML2B |
| NMI | OLFML3 |
| NMNAT2 | OMD |
| NMRAL1 | OPTC |
| NMU | OR11A1 |
| NOS1AP | OR11G2 |
| NOXA1 | OR1J1 |
| NOXO1 | OR1J4 |
| NPAS1 | OR2A2 |
| NPAS2 | OR2T8 |
| NPCDR1 | OR51B6 |
| NPHP3 | OR5F1 |
| NPRL3 | OR5P2 |
| NQO1 | OR8J1 |
| NR0B1 | OSBPL1A |
| NR0B2 | OSBPL5 |
| NR1I2 | OSBPL6 |
| NR2C2AP | OSM |
| NR2E3 | OSR1 |
| NR2F6 | OSR2 |
| NRL | OSTM1 |
| NRSN2 | OXCT1 |
| NRTN | OXCT2 |
| NSDHL | P2RX1 |
| NSG1 | P3H3 |
| NSUN5 | P4HA3 |
| NSUN5P1 | PADI4 |
| NSUN7 | PAG1 |
| NTF4 | PAGE4 |
| NUDT13 | PAIP2 |
| NUDT14 | PAK3 |
| NUDT15 | PALLD |
| NUDT16L1 | PALM |
| NUDT19 | PALMD |
| NUDT22 | PAM |
| NUDT5 | PAMR1 |
| NUDT8 | PANX1 |
| NUF2 | PAPPA |
| NUP210 | PAPSS2 |
| NUP43 | PAQR3 |
| NUP62 | PARM1 |
| NUP62CL | PARP15 |
| NUSAP1 | PARVB |
| NXF3 | PBRM1 |
| OAS1 | PBX3 |
| OAS3 | PCDH11Y |
| OBP2A | PCDH12 |
| OBSCN | PCDH18 |
| OCIAD2 | PCDH20 |
| OCLM | PCDH7 |
| OCLN | PCDHB10 |
| OFD1 | PCDHB11 |
| OGDHL | PCDHB12 |
| OGFOD2 | PCDHB14 |
| OIP5 | PCDHB15 |
| OPLAH | PCDHB16 |
| OPN3 | PCDHB4 |
| OR10K2 | PCDHB9 |
| OR1G1 | PCDHGA8 |
| OR1L3 | PCOLCE |
| OR2A9P | PCOLCE2 |
| OR2L3 | PCP4 |
| OR4D5 | PCSK5 |
| OR52B2 | PDE1A |
| OR5C1 | PDE1C |
| OR5T1 | PDE2A |
| OR7C2 | PDE3B |
| OR7E19P | PDE4B |
| OR7E24 | PDE4D |
| OR7E5P | PDE5A |
| OR7E91P | PDE8B |
| OR8B8 | PDGFA |
| OR8K5 | PDGFD |
| OR8S1 | PDGFRA |
| ORC1 | PDGFRB |
| ORC4 | PDGFRL |
| ORC5 | PDLIM2 |
| ORC6 | PDLIM3 |
| ORMDL2 | PDLIM4 |
| OSBP2 | PDLIM5 |
| OSBPL3 | PDLIM7 |
| OTP | PDPN |
| OTUB2 | PDS5B |
| OTUD6B | PDZRN3 |
| OTX1 | PEAK1 |
| OVOL1 | PECAM1 |
| OVOL2 | PELO |
| OXLD1 | PER1 |
| OXNAD1 | PER3 |
| OXSM | PF4 |
| P2RX4 | PGAP1 |
| P2RY2 | PGBD1 |
| P2RY6 | PGF |
| PA2G4 | PGM5 |
| PACSIN1 | PGR |
| PAFAH1B3 | PHF13 |
| PAGE5 | PHF14 |
| PAICS | PHF19 |
| PAIP1 | PHLDA1 |
| PAK1 | PHLDB1 |
| PAK4 | PHLDB2 |
| PAM16 | PHYHIP |
| PANK1 | PI15 |
| PAQR4 | PI16 |
| PAQR6 | PICALM |
| PARD6B | PID1 |
| PARP1 | PIEZO1 |
| PARP12 | PIEZO2 |
| PARP4 | PIK3C2B |
| PARPBP | PIK3C2G |
| PARS2 | PIM1 |
| PART1 | PINK1 |
| PASK | PITPNM3 |
| PATJ | PJA2 |
| PATZ1 | PKD1 |
| PAX1 | PKD2 |
| PAX8 | PKIA |
| PAXIP1 | PKIG |
| PBK | PKNOX2 |
| PBOV1 | PLA2G2A |
| PBX4 | PLA2G4A |
| PC | PLA2G4C |
| PCBD1 | PLA2G5 |
| PCBD2 | PLA2R1 |
| PCCA | PLAGL1 |
| PCCB | PLAT |
| PCDHB2 | PLAU |
| PCDHB8 | PLAUR |
| PCED1B | PLCD4 |
| PCK1 | PLCG2 |
| PCK2 | PLCL1 |
| PCNA | PLCL2 |
| PDCD2L | PLEK |
| PDCD5 | PLEKHA4 |
| PDE6B | PLEKHO1 |
| PDE6G | PLEKHO2 |
| PDE7B | PLIN1 |
| PDHA1 | PLIN2 |
| PDIA2 | PLIN4 |
| PDIA4 | PLK3 |
| PDIA6 | PLN |
| PDIK1L | PLP2 |
| PDK1 | PLPP1 |
| PDRG1 | PLPP3 |
| PDXK | PLPP7 |
| PDZK1IP1 | PLPPR4 |
| PECR | PLS3 |
| PEG10 | PLSCR4 |
| PERP | PLVAP |
| PEX11A | PLXDC1 |
| PEX11B | PLXNA2 |
| PFDN6 | PMEPA1 |
| PFN4 | PMP2 |
| PGAP2 | PMP22 |
| PGBD3 | PNMA1 |
| PGK1 | PNPLA7 |
| PGLS | PNPLA8 |
| PGLYRP4 | PNRC1 |
| PHACTR3 | PNRC2 |
| PHF8 | PODN |
| PHGDH | POLK |
| PHKG2 | POLR2M |
| PHLDA2 | POLR3GL |
| PIAS3 | POMGNT2 |
| PIGO | POPDC2 |
| PIGR | POSTN |
| PIH1D2 | POU4F3 |
| PIK3AP1 | POU6F1 |
| PIK3IP1 | PPARG |
| PIK3R2 | PPARGC1A |
| PILRA | PPBP |
| PIP4K2B | PPFIA2 |
| PITPNM1 | PPIC |
| PITX1 | PPM1D |
| PIWIL1 | PPM1K |
| PKD2L1 | PPM1M |
| PKM | PPP1R12A |
| PKMYT1 | PPP1R12C |
| PKP1 | PPP1R14A |
| PKP2 | PPP1R15A |
| PKP3 | PPP1R15B |
| PKP4 | PPP1R16B |
| PLA2G10 | PPP1R17 |
| PLA2G12A | PPP1R3A |
| PLA2G4E | PPP1R3B |
| PLAC1 | PPP1R3C |
| PLAG1 | PPP2CB |
| PLAGL2 | PPP2R2B |
| PLEK2 | PPP2R3C |
| PLEKHB1 | PPP3CC |
| PLEKHB2 | PRDM2 |
| PLEKHG4B | PRELP |
| PLEKHN1 | PRG4 |
| PLEKHS1 | PRICKLE1 |
| PLK1 | PRKAG2 |
| PLK4 | PRKAR1A |
| PLLP | PRKCB |
| PLPP2 | PRKCDBP |
| PLPP5 | PRKCH |
| PLS1 | PRKD1 |
| PLXNB1 | PRKG1 |
| PMAIP1 | PRLR |
| PNKP | PRM2 |
| PNLIP | PRNP |
| PNMAL1 | PROCR |
| PNOC | PROK2 |
| PNPLA3 | PROS1 |
| PNPLA4 | PRPH |
| PNPT1 | PRPS1 |
| POC1A | PRR16 |
| POC1B | PRR27 |
| PODXL2 | PRRT2 |
| POF1B | PRRX1 |
| POLA2 | PRSS23 |
| POLD1 | PRSS35 |
| POLD2 | PRX |
| POLE | PSAP |
| POLE2 | PSTPIP1 |
| POLQ | PTCHD1 |
| POLR2H | PTGDR |
| POMGNT1 | PTGDS |
| PON2 | PTGER3 |
| POP1 | PTGER4 |
| POP5 | PTGFR |
| POR | PTGIR |
| POU3F1 | PTGIS |
| POU5F1 | PTGS2 |
| PPA1 | PTN |
| PPARGC1B | PTPN13 |
| PPAT | PTPN5 |
| PPDPF | PTPRB |
| PPEF2 | PTPRC |
| PPFIBP1 | PTPRD |
| PPFIBP2 | PTPRE |
| PPIF | PTPRN2 |
| PPIL2 | PTPRO |
| PPIL6 | PTPRZ1 |
| PPL | PTRF |
| PPM1G | PTX3 |
| PPM1H | PXDN |
| PPM1J | PYGM |
| PPP1CA | PZP |
| PPP1R14B | QKI |
| PPP1R14C | R3HDML |
| PPP1R16A | RAB11FIP2 |
| PPP1R1B | RAB11FIP5 |
| PPP1R26 | RAB23 |
| PPP1R35 | RAB30 |
| PPP1R9A | RAB31 |
| PPP4C | RAB32 |
| PRAME | RAB33A |
| PRC1 | RAB3C |
| PRDX2 | RAB3IL1 |
| PRDX5 | RAB40A |
| PRELID3B | RAB7B |
| PREPL | RAB8B |
| PRICKLE3 | RAB9B |
| PRIM1 | RABEP1 |
| PRKAA2 | RABGAP1L |
| PRKAB1 | RAD51B |
| PRKCD | RADIL |
| PRKCI | RAI14 |
| PRKCQ | RAMP1 |
| PRKCSH | RAMP2 |
| PRKCZ | RAMP3 |
| PRKX | RAN |
| PRMT6 | RANBP6 |
| PRODH | RAP1A |
| PROM1 | RAP1B |
| PROM2 | RAP1GDS1 |
| PROSER1 | RAP2C |
| PROSER2 | RAPGEF4 |
| PRR13 | RAPGEF5 |
| PRR15 | RASA4 |
| PRR15L | RASD1 |
| PRR7 | RASGRF2 |
| PRRG2 | RASGRP3 |
| PRRG4 | RASIP1 |
| PRRT3 | RASL11A |
| PRSS1 | RASL11B |
| PRSS16 | RASSF2 |
| PRSS2 | RASSF8 |
| PRSS21 | RBM7 |
| PRSS22 | RBMS1 |
| PRSS3 | RBMS3 |
| PRSS33 | RBP5 |
| PRSS50 | RBP7 |
| PRSS8 | RBPMS |
| PSAT1 | RBPMS2 |
| PSCA | RCAN1 |
| PSD4 | RCAN2 |
| PSENEN | RCHY1 |
| PSMB10 | RCN3 |
| PSMC4 | RCSD1 |
| PSME1 | RECK |
| PSME2 | RECQL5 |
| PSMG3 | REEP1 |
| PSPH | REM1 |
| PSPHP1 | RERG |
| PSRC1 | RERGL |
| PTBP3 | REV3L |
| PTCD1 | REXO2 |
| PTER | RFX2 |
| PTGR2 | RGCC |
| PTGS1 | RGL1 |
| PTH2R | RGL4 |
| PTK6 | RGMA |
| PTPN14 | RGN |
| PTPN3 | RGS16 |
| PTPN4 | RGS18 |
| PTPN6 | RGS2 |
| PTPRF | RGS22 |
| PTPRJ | RGS3 |
| PTPRS | RGS4 |
| PTPRU | RGS5 |
| PTTG1 | RGS9 |
| PTTG2 | RHOA |
| PUF60 | RHOBTB1 |
| PUS10 | RHOBTB3 |
| PXMP4 | RHOH |
| PYCR1 | RHOJ |
| PYCRL | RILPL2 |
| PYGO2 | RIMS3 |
| QARS | RIPK1 |
| QPRT | RND1 |
| RAB11FIP4 | RND3 |
| RAB17 | RNF11 |
| RAB25 | RNF115 |
| RAB26 | RNF122 |
| RAB36 | RNF125 |
| RAB3D | RNF130 |
| RAB3IP | RNF146 |
| RAB40C | RNF180 |
| RAB42 | RNF19B |
| RAB43 | ROBO3 |
| RAC3 | ROBO4 |
| RACGAP1 | ROCK1 |
| RACGAP1P | ROCK2 |
| RAD21 | ROR1 |
| RAD51 | ROR2 |
| RAD51AP1 | RORA |
| RAD54B | RPGRIP1 |
| RAD54L | RPL15 |
| RAE1 | RPL23AP32 |
| RALGPS1 | RPRM |
| RALGPS2 | RPS6KA3 |
| RANBP17 | RRAD |
| RANGAP1 | RRAS |
| RAP1GAP | RSPO1 |
| RAPGEFL1 | RSPO3 |
| RARRES3 | RSU1 |
| RASAL1 | RTN4 |
| RASGEF1A | RUFY3 |
| RASGRF1 | RUNDC3B |
| RASGRP1 | RUNX1T1 |
| RASSF6 | RUSC2 |
| RASSF7 | RWDD1 |
| RBFOX1 | RXFP2 |
| RBKS | RYBP |
| RBM11 | RYR3 |
| RBM15 | S100A12 |
| RBM33 | S100A3 |
| RBM41 | S100B |
| RBM47 | S1PR1 |
| RBMXL3 | S1PR4 |
| RCAN3 | S1PR5 |
| RCC1 | SACS |
| RCC2 | SAMD3 |
| RCCD1 | SAMD4A |
| RDH12 | SAMSN1 |
| RDH13 | SAP30 |
| RDM1 | SARAF |
| REC8 | SASH1 |
| RECQL4 | SATB1 |
| REG1B | SBDS |
| REPIN1 | SBSPON |
| REST | SCARA5 |
| REXO4 | SCARB2 |
| RFC3 | SCARF2 |
| RFC4 | SCHIP1 |
| RFC5 | SCN2B |
| RFWD3 | SCN4B |
| RFXANK | SCN7A |
| RGL3 | SCN9A |
| RGP1 | SCRG1 |
| RGS20 | SDC2 |
| RGS6 | SDC3 |
| RHBDD2 | SDCBP |
| RHBDD3 | SDPR |
| RHBDF2 | SDR9C7 |
| RHBDL2 | SEC23A |
| RHCE | SEC24D |
| RHNO1 | SEC62 |
| RHOD | SELE |
| RHOF | SELL |
| RHOV | SELM |
| RHOXF1 | SELP |
| RHPN1 | SEMA3C |
| RHPN2 | SEMA3G |
| RIBC1 | SEMA5A |
| RIBC2 | SEMG1 |
| RIDA | SEPP1 |
| RIMS2 | SEPW1 |
| RIPK4 | SERINC1 |
| RIPPLY3 | SERP2 |
| RITA1 | SERPINB10 |
| RLN2 | SERPINB6 |
| RMI1 | SERPIND1 |
| RMI2 | SERPINE2 |
| RMND5A | SERPINF1 |
| RNASEH2A | SERPING1 |
| RNASET2 | SERPINH1 |
| RNF183 | SERPINI1 |
| RNF186 | SERTAD1 |
| RNF39 | SESN1 |
| RNF43 | SETBP1 |
| RNFT2 | SETD7 |
| ROMO1 | SFRP1 |
| RORC | SFRP2 |
| RPA3 | SFRP4 |
| RPE | SFXN3 |
| RPP25L | SGCA |
| RPRD1B | SGCB |
| RPS2P32 | SGCD |
| RPS6KA1 | SGCE |
| RPUSD1 | SGIP1 |
| RPUSD2 | SGK1 |
| RPUSD3 | SGPP1 |
| RRAGD | SH2D1B |
| RRM2 | SH2D3C |
| RRP1 | SH3BGR |
| RRP9 | SH3BGRL |
| RSAD2 | SH3BP4 |
| RSPH1 | SH3BP5 |
| RSPH10B | SH3D19 |
| RSPH14 | SH3PXD2A |
| RSPH3 | SH3PXD2B |
| RSRP1 | SHC3 |
| RTKN | SHISA4 |
| RTKN2 | SHOC2 |
| RTP4 | SI |
| RUVBL1 | SIK1 |
| RXFP1 | SIK2 |
| S100A1 | SKI |
| S100A11 | SLAMF1 |
| S100A14 | SLC12A4 |
| S100A2 | SLC12A5 |
| S100A5 | SLC16A2 |
| SAA1 | SLC16A7 |
| SAA2 | SLC19A2 |
| SAA4 | SLC1A7 |
| SAC3D1 | SLC22A11 |
| SALL4 | SLC22A17 |
| SAMD9 | SLC22A2 |
| SARS2 | SLC22A3 |
| SAXO1 | SLC24A3 |
| SCD | SLC25A25 |
| SCEL | SLC25A32 |
| SCGB1D1 | SLC2A3 |
| SCGB1D2 | SLC34A1 |
| SCGB1D4 | SLC35G2 |
| SCGB2A1 | SLC39A14 |
| SCIN | SLC43A1 |
| SCN1A | SLC45A3 |
| SCNN1A | SLC47A1 |
| SCRIB | SLC6A2 |
| SCRN1 | SLC9B2 |
| SCRT1 | SLCO2A1 |
| SDC1 | SLCO2B1 |
| SDF2L1 | SLFN11 |
| SDHAF4 | SLIT2 |
| SDSL | SLIT3 |
| SEC14L4 | SLITRK3 |
| SEMA4B | SLMAP |
| SEMA4D | SMAD4 |
| SEMA4G | SMAD5-AS1 |
| SENP8 | SMAD7 |
| SEPSECS | SMAD9 |
| SERINC2 | SMARCA1 |
| SERPINA1 | SMARCA2 |
| SERPINA5 | SMG6 |
| SERPINA9 | SMIM3 |
| SERPINB4 | SMOC2 |
| SERPINB9 | SMPDL3A |
| SERPINI2 | SMTN |
| SETD6 | SNAI1 |
| SETDB1 | SNAI2 |
| SEZ6L2 | SNAP23 |
| SFN | SNCA |
| SFT2D3 | SNCAIP |
| SFTA2 | SNRK |
| SFTPD | SNTG2 |
| SFXN1 | SNX18 |
| SFXN2 | SNX21 |
| SGK2 | SNX9 |
| SGO1 | SOAT2 |
| SGO2 | SOBP |
| SGPL1 | SOCS2 |
| SGPP2 | SOCS3 |
| SH2D3A | SOCS5 |
| SH2D4A | SOD3 |
| SH3BP2 | SORBS1 |
| SH3D21 | SORBS2 |
| SH3GL3 | SORBS3 |
| SH3RF2 | SORCS2 |
| SH3YL1 | SOX7 |
| SHANK2 | SOX8 |
| SHCBP1 | SPARC |
| SHH | SPARCL1 |
| SHMT1 | SPATA3 |
| SHMT2 | SPECC1 |
| SHROOM2 | SPG20 |
| SHTN1 | SPHAR |
| SIGLEC10 | SPHK1 |
| SIGLEC15 | SPOCD1 |
| SIM2 | SPOCK1 |
| SIX1 | SPON2 |
| SIX6 | SPOP |
| SKA3 | SPRED1 |
| SKAP1 | SPRY1 |
| SLAIN1 | SPRYD3 |
| SLAMF8 | SPSB1 |
| SLC11A2 | SPTBN1 |
| SLC12A8 | SPTBN4 |
| SLC13A2 | SPX |
| SLC13A5 | SRC |
| SLC15A2 | SRF |
| SLC16A13 | SRGN |
| SLC16A3 | SRI |
| SLC16A5 | SRPX |
| SLC16A6 | SRPX2 |
| SLC17A3 | SRR |
| SLC1A3 | SRSF5 |
| SLC1A6 | SSPN |
| SLC22A18 | SSTR1 |
| SLC25A10 | SSTR2 |
| SLC25A13 | SSUH2 |
| SLC25A15 | SSX5 |
| SLC25A16 | ST3GAL3 |
| SLC25A22 | ST3GAL5 |
| SLC25A29 | ST5 |
| SLC25A39 | ST6GALNAC3 |
| SLC25A40 | ST6GALNAC6 |
| SLC26A2 | ST7L |
| SLC26A9 | ST8SIA1 |
| SLC27A2 | STAB1 |
| SLC27A6 | STAB2 |
| SLC28A1 | STARD13 |
| SLC28A2 | STARD5 |
| SLC2A1 | STARD6 |
| SLC30A2 | STARD8 |
| SLC30A6 | STAT4 |
| SLC34A2 | STAT5A |
| SLC35F2 | STBD1 |
| SLC35F3 | STEAP4 |
| SLC35F5 | STK10 |
| SLC37A1 | STK26 |
| SLC37A4 | STK38L |
| SLC38A1 | STK40 |
| SLC39A10 | STOM |
| SLC39A4 | STX11 |
| SLC3A1 | STX12 |
| SLC44A3 | STX2 |
| SLC44A4 | STX7 |
| SLC46A1 | STXBP5 |
| SLC4A11 | SULF1 |
| SLC50A1 | SULF2 |
| SLC51A | SV2B |
| SLC51B | SVIL |
| SLC52A2 | SWAP70 |
| SLC52A3 | SYCP3 |
| SLC5A1 | SYDE1 |
| SLC5A11 | SYNC |
| SLC5A3 | SYNDIG1 |
| SLC5A6 | SYNE1 |
| SLC6A12 | SYNGR1 |
| SLC6A14 | SYNJ2 |
| SLC6A20 | SYNM |
| SLC6A6 | SYNPO |
| SLC7A11 | SYT1 |
| SLC7A9 | SYT11 |
| SLC9A3R1 | SYTL4 |
| SLC9A7 | TAC1 |
| SLC9A8 | TACC1 |
| SLFN13 | TACC2 |
| SLITRK2 | TACR2 |
| SLPI | TAGAP |
| SLURP1 | TAGLN |
| SLX1A | TAL1 |
| SMARCC1 | TBC1D1 |
| SMC1B | TBC1D2B |
| SMC2 | TBC1D8B |
| SMC5 | TBRG1 |
| SMCO2 | TBX1 |
| SMG7 | TBX18 |
| SMPD2 | TBX2 |
| SMPD3 | TBX3 |
| SMPDL3B | TBXA2R |
| SMUG1 | TCEAL1 |
| SNCB | TCEAL2 |
| SNRPA | TCEAL4 |
| SNRPA1 | TCEAL5 |
| SNRPB | TCEAL7 |
| SNRPC | TCF15 |
| SNTB1 | TCF21 |
| SORD | TCF4 |
| SORL1 | TCF7L1 |
| SORT1 | TCP11L1 |
| SOX12 | TCTEX1D1 |
| SOX2 | TDGF1 |
| SOX21 | TEK |
| SOX3 | TENM3 |
| SOX4 | TEX14 |
| SOX9 | TEX29 |
| SP1 | TFE3 |
| SP140L | TFF3 |
| SPA17 | TFPI |
| SPAG11B | TGFB1 |
| SPAG17 | TGFB1I1 |
| SPAG4 | TGFB2 |
| SPAG5 | TGFB3 |
| SPAG8 | TGFBI |
| SPATA17 | TGFBR1 |
| SPATA9 | TGFBR2 |
| SPC25 | TGFBR3 |
| SPDEF | THBD |
| SPDL1 | THBS1 |
| SPHK2 | THBS2 |
| SPICE1 | THBS4 |
| SPINK1 | THEG |
| SPINK5 | THRA |
| SPINT1 | THY1 |
| SPINT2 | TIE1 |
| SPIRE2 | TIMP3 |
| SPOCK2 | TIMP4 |
| SPP1 | TINAGL1 |
| SPR | TIPARP |
| SPRR1A | TLE2 |
| SPRR1B | TLE4 |
| SPRY3 | TLL1 |
| SPSB2 | TLN1 |
| SPTBN2 | TLR1 |
| SQLE | TLR4 |
| SRD5A1 | TLR8 |
| SRD5A3 | TM4SF1 |
| SRGAP3 | TM4SF18 |
| SRPK1 | TM6SF1 |
| SSC4D | TMEM109 |
| SST | TMEM119 |
| SSX3 | TMEM133 |
| SSX7 | TMEM150C |
| ST14 | TMEM158 |
| ST3GAL6 | TMEM159 |
| ST6GALNAC1 | TMEM173 |
| ST6GALNAC2 | TMEM200A |
| ST8SIA5 | TMEM204 |
| STAC | TMEM255A |
| STAG3 | TMEM263 |
| STAG3L3 | TMEM35A |
| STAP2 | TMEM43 |
| STAR | TMEM45A |
| STAT1 | TMEM47 |
| STC2 | TMEM55A |
| STEAP3 | TMEM59L |
| STK31 | TMEM70 |
| STMN1 | TMEM71 |
| STON2 | TMEM88 |
| STOX1 | TMEM98 |
| STRA6 | TMSB15B |
| STRADB | TNC |
| STRBP | TNFAIP3 |
| STRC | TNFAIP6 |
| STX16 | TNFRSF10C |
| STXBP2 | TNFRSF8 |
| STXBP6 | TNFSF12 |
| STYK1 | TNNC2 |
| STYXL1 | TNNT3 |
| SUGP1 | TNS1 |
| SULT1C2 | TNS2 |
| SULT1C4 | TNXB |
| SURF2 | TOM1L2 |
| SUSD4 | TOR1AIP1 |
| SUV39H1 | TP53AIP1 |
| SVOPL | TPM1 |
| SWSAP1 | TPM2 |
| SYBU | TPM4 |
| SYK | TPO |
| SYNE2 | TPPP3 |
| SYNGR2 | TPSAB1 |
| SYNGR3 | TRAF3IP2 |
| SYT13 | TRAF5 |
| SYT17 | TREM1 |
| SYT8 | TRIB1 |
| SYTL1 | TRIM4 |
| TACC3 | TRIM7 |
| TACSTD2 | TRO |
| TAF4 | TRPC1 |
| TALDO1 | TRPC4 |
| TAPBPL | TRPC5 |
| TARBP2 | TRPC6 |
| TARS2 | TRPM6 |
| TAS1R2 | TRPV2 |
| TAS2R10 | TSC22D1 |
| TAS2R31 | TSC22D2 |
| TAS2R38 | TSC22D3 |
| TAS2R5 | TSHZ2 |
| TBC1D2 | TSHZ3 |
| TBC1D31 | TSKS |
| TBC1D3F | TSLP |
| TBL1XR1 | TSPAN11 |
| TBRG4 | TSPAN18 |
| TCF7 | TSPAN2 |
| TCP11L2 | TSPAN4 |
| TCTEX1D2 | TSPAN5 |
| TDP1 | TSPAN7 |
| TDRKH | TSPAN9 |
| TEC | TSPYL2 |
| TECR | TSPYL5 |
| TEKT2 | TTC28 |
| TENM4 | TTC6 |
| TERT | TTC7B |
| TESMIN | TTL |
| TEX10 | TTLL7 |
| TFAP2A | TTR |
| TFAP2B | TUB |
| TFAP2C | TUBB2A |
| TFCP2L1 | TUBB6 |
| TFDP2 | TUBE1 |
| TFF2 | TULP2 |
| TFR2 | TWIST1 |
| TGFA | TWIST2 |
| TGM1 | TWSG1 |
| TGM3 | TXLNB |
| TGM4 | TXNDC15 |
| TGOLN2 | TXNDC2 |
| THEM6 | TXNRD1 |
| THOC3 | UAP1 |
| TICRR | UBB |
| TIGD2 | UBE2E2 |
| TIGD5 | UBE2Q2 |
| TIMD4 | UBL3 |
| TIMELESS | UBR1 |
| TIMM17B | UFM1 |
| TK1 | UFSP2 |
| TKT | ULK2 |
| TKTL2 | UNC5C |
| TLCD1 | UPB1 |
| TLE1 | USHBP1 |
| TLX3 | USO1 |
| TM7SF2 | USP12 |
| TMBIM6 | USP15 |
| TMC4 | USP31 |
| TMC5 | UST |
| TMC6 | VAMP3 |
| TMCO6 | VAMP5 |
| TMEM102 | VAPA |
| TMEM125 | VASH1 |
| TMEM132A | VAT1 |
| TMEM139 | VCAM1 |
| TMEM141 | VCAN |
| TMEM155 | VEGFC |
| TMEM164 | VENTX |
| TMEM177 | VGLL3 |
| TMEM186 | VIM |
| TMEM187 | VIPR2 |
| TMEM205 | VIT |
| TMEM209 | VKORC1 |
| TMEM223 | VNN2 |
| TMEM254 | VSX1 |
| TMEM260 | VWF |
| TMEM266 | WBP4 |
| TMEM267 | WBSCR17 |
| TMEM27 | WDR44 |
| TMEM33 | WDR47 |
| TMEM38A | WFDC1 |
| TMEM41A | WFS1 |
| TMEM45B | WIPF1 |
| TMEM61 | WIPI1 |
| TMEM68 | WISP1 |
| TMEM69 | WISP2 |
| TMEM74 | WNT2 |
| TMEM79 | WNT2B |
| TMEM8A | WNT4 |
| TMEM92 | WNT5B |
| TMIE | WNT8B |
| TMIGD3 | WRB |
| TMLHE | WSB1 |
| TMPO | WSCD1 |
| TMPRSS2 | WTAP |
| TMPRSS4 | XG |
| TMPRSS6 | XPNPEP2 |
| TMTC4 | YPEL4 |
| TMUB1 | ZBTB16 |
| TNF | ZBTB20 |
| TNFRSF11B | ZBTB38 |
| TNFRSF21 | ZBTB4 |
| TNFRSF9 | ZC4H2 |
| TNFSF10 | ZCCHC12 |
| TNFSF13 | ZCCHC24 |
| TNK1 | ZDHHC2 |
| TNNC1 | ZEB1 |
| TNNI2 | ZEB2 |
| TNNI3 | ZFHX4 |
| TNNT1 | ZFP2 |
| TNS4 | ZFP36 |
| TOM1L1 | ZFP36L1 |
| TOMM34 | ZFP37 |
| TOMM40 | ZFP82 |
| TONSL | ZFPM2 |
| TOP1MT | ZFYVE16 |
| TOP2A | ZIC4 |
| TOPBP1 | ZMAT3 |
| TOR2A | ZNF10 |
| TP53TG3 | ZNF106 |
| TPD52 | ZNF175 |
| TPD52L1 | ZNF219 |
| TPGS2 | ZNF222 |
| TPI1 | ZNF25 |
| TPX2 | ZNF280D |
| TRAF2 | ZNF331 |
| TRAF4 | ZNF333 |
| TRAIP | ZNF385B |
| TRAP1 | ZNF385D |
| TRAPPC9 | ZNF410 |
| TREH | ZNF423 |
| TREM2 | ZNF436 |
| TREX1 | ZNF438 |
| TRHR | ZNF449 |
| TRIB3 | ZNF516 |
| TRIM11 | ZNF521 |
| TRIM14 | ZNF548 |
| TRIM17 | ZNF575 |
| TRIM24 | ZNF689 |
| TRIM29 | ZNF75D |
| TRIM31 | ZNF781 |
| TRIM36 | ZXDA |
| TRIM45 | ZYX |
| TRIM51 |  |
| TRIM52 |  |
| TRIP13 |  |
| TRMT2A |  |
| TROAP |  |
| TRPM5 |  |
| TRPV1 |  |
| TRPV6 |  |
| TRUB2 |  |
| TSEN54 |  |
| TSFM |  |
| TSGA10 |  |
| TSPAN1 |  |
| TSSC1 |  |
| TST |  |
| TSTA3 |  |
| TSTD1 |  |
| TTC12 |  |
| TTC16 |  |
| TTC23L |  |
| TTC30A |  |
| TTC30B |  |
| TTC32 |  |
| TTC39A |  |
| TTC39C |  |
| TTC9 |  |
| TTF2 |  |
| TTI1 |  |
| TTK |  |
| TTLL12 |  |
| TTLL4 |  |
| TUBAL3 |  |
| TUBB |  |
| TUBG1 |  |
| TUBGCP4 |  |
| TUFM |  |
| TUFT1 |  |
| TUT1 |  |
| TVP23C |  |
| TXK |  |
| TXNDC17 |  |
| TYMS |  |
| TYSND1 |  |
| UBAP2 |  |
| UBD |  |
| UBE2C |  |
| UBE2T |  |
| UBFD1 |  |
| UBXN10 |  |
| UBXN11 |  |
| UCP2 |  |
| UGGT1 |  |
| UGT2B10 |  |
| UGT2B11 |  |
| UGT2B7 |  |
| UGT8 |  |
| UHRF1 |  |
| UHRF1BP1 |  |
| UMPS |  |
| UNC13D |  |
| UNC5CL |  |
| UNC93B1 |  |
| UNG |  |
| UPF1 |  |
| UPK1B |  |
| UPK3A |  |
| UPK3B |  |
| UQCR10 |  |
| USP18 |  |
| USP21 |  |
| USP28 |  |
| USP43 |  |
| USP45 |  |
| USP54 |  |
| USP6NL |  |
| VAC14 |  |
| VAMP8 |  |
| VARS |  |
| VARS2 |  |
| VAV3 |  |
| VAX2 |  |
| VCX2 |  |
| VCX3A |  |
| VCX3B |  |
| VCY |  |
| VEPH1 |  |
| VGF |  |
| VGLL1 |  |
| VMA21 |  |
| VPS33B |  |
| VPS37D |  |
| VPS53 |  |
| VSTM2A |  |
| VTCN1 |  |
| VWA3A |  |
| WARS2 |  |
| WBSCR27 |  |
| WDHD1 |  |
| WDR12 |  |
| WDR18 |  |
| WDR34 |  |
| WDR5 |  |
| WDR54 |  |
| WDR5B |  |
| WDR62 |  |
| WDR66 |  |
| WDR78 |  |
| WDR90 |  |
| WDR91 |  |
| WHSC1 |  |
| WHSC1L1 |  |
| WNT10A |  |
| WNT5A |  |
| WNT7A |  |
| WRNIP1 |  |
| WWC1 |  |
| XAGE2 |  |
| XAGE5 |  |
| XCL2 |  |
| XDH |  |
| XPO1 |  |
| XPR1 |  |
| XRN1 |  |
| YARS2 |  |
| YBX2 |  |
| YPEL1 |  |
| ZADH2 |  |
| ZBED2 |  |
| ZBED6CL |  |
| ZBP1 |  |
| ZBTB10 |  |
| ZBTB12 |  |
| ZBTB26 |  |
| ZBTB37 |  |
| ZC2HC1C |  |
| ZC3H3 |  |
| ZDHHC12 |  |
| ZDHHC13 |  |
| ZDHHC16 |  |
| ZDHHC20 |  |
| ZDHHC21 |  |
| ZDHHC23 |  |
| ZDHHC9 |  |
| ZFP41 |  |
| ZFP64 |  |
| ZG16B |  |
| ZIC1 |  |
| ZIC2 |  |
| ZMYM3 |  |
| ZMYND10 |  |
| ZMYND12 |  |
| ZMYND8 |  |
| ZNF101 |  |
| ZNF107 |  |
| ZNF117 |  |
| ZNF138 |  |
| ZNF16 |  |
| ZNF165 |  |
| ZNF185 |  |
| ZNF195 |  |
| ZNF205-AS1 |  |
| ZNF208 |  |
| ZNF217 |  |
| ZNF239 |  |
| ZNF250 |  |
| ZNF273 |  |
| ZNF280A |  |
| ZNF280B |  |
| ZNF296 |  |
| ZNF3 |  |
| ZNF320 |  |
| ZNF396 |  |
| ZNF429 |  |
| ZNF43 |  |
| ZNF430 |  |
| ZNF431 |  |
| ZNF433 |  |
| ZNF440 |  |
| ZNF441 |  |
| ZNF442 |  |
| ZNF443 |  |
| ZNF467 |  |
| ZNF48 |  |
| ZNF485 |  |
| ZNF486 |  |
| ZNF492 |  |
| ZNF493 |  |
| ZNF497 |  |
| ZNF506 |  |
| ZNF518A |  |
| ZNF536 |  |
| ZNF544 |  |
| ZNF549 |  |
| ZNF552 |  |
| ZNF556 |  |
| ZNF560 |  |
| ZNF563 |  |
| ZNF57 |  |
| ZNF572 |  |
| ZNF581 |  |
| ZNF587 |  |
| ZNF589 |  |
| ZNF600 |  |
| ZNF606 |  |
| ZNF610 |  |
| ZNF618 |  |
| ZNF623 |  |
| ZNF625 |  |
| ZNF627 |  |
| ZNF646 |  |
| ZNF664 |  |
| ZNF675 |  |
| ZNF678 |  |
| ZNF681 |  |
| ZNF682 |  |
| ZNF683 |  |
| ZNF687 |  |
| ZNF691 |  |
| ZNF692 |  |
| ZNF701 |  |
| ZNF702P |  |
| ZNF707 |  |
| ZNF714 |  |
| ZNF74 |  |
| ZNF750 |  |
| ZNF772 |  |
| ZNF774 |  |
| ZNF782 |  |
| ZNF786 |  |
| ZNF816 |  |
| ZNF823 |  |
| ZNF839 |  |
| ZNF85 |  |
| ZNF91 |  |
| ZNHIT1 |  |
| ZNHIT2 |  |
| ZP3 |  |
| ZPBP |  |
| ZPBP2 |  |
| ZSCAN16 |  |
| ZSCAN2 |  |
| ZSCAN25 |  |
| ZSCAN29 |  |
| ZSWIM3 |  |
| ZWILCH |  |
