# Supplementary material for: Dysregulation of pseudogene/lncRNA-hsa-miR-363-3p-SPOCK2 pathway fuels stage progression of ovarian cancer
Source: Aging (Albany NY). 2019 Dec 3;11(23):11416–39. doi: 10.18632/aging.102538 (PMC6932902; doi:10.18632/aging.102538)
Supplement: Supplementary Table 2 [file aging-11-102538-s001..docx]

**Supplementary Table 2. The significant DEGs between early ovarian cancer samples and advanced ovarian cancer samples.**

| Upregulated DEGs in advanced ovarian cancer samples | Downregulated DEGs in advanced ovarian cancer samples |
| --- | --- |
| AADACL2 | ACE2 |
| AADACL4 | ACSBG1 |
| ABCA6 | ACSM3 |
| ABHD1 | ACTRT2 |
| ACKR3 | ADGRF2 |
| ACSM5 | ADGRF4 |
| ACSS3 | AGR2 |
| ACTA2 | AK7 |
| ACTG2 | AKNAD1 |
| ADAM12 | AMIGO1 |
| ADAM19 | ANK1 |
| ADAM8 | AOC1 |
| ADAMDEC1 | ARRDC4 |
| ADAMTS2 | ARSE |
| ADAMTSL1 | ART3 |
| ADARB1 | ATOH1 |
| ADGRE5 | ATP2B3 |
| ADGRG2 | ATP6V1C2 |
| ADGRL2 | B3GNT4 |
| ADIPOQ | B3GNT7 |
| ADRA1B | BCO1 |
| AEBP1 | C3orf49 |
| AFAP1L2 | C4orf19 |
| AFF3 | C7orf62 |
| AGBL1 | C8orf31 |
| AGXT | C9orf66 |
| AK5 | CA12 |
| AKAP17A | CAPN13 |
| ALDH1A3 | CD177 |
| ALDOC | CDH10 |
| ALOX5AP | CDH16 |
| ANG | CEACAM1 |
| ANKH | CELF3 |
| ANTXR1 | CEP44 |
| ANTXR2 | CFAP47 |
| ANXA13 | CHIA |
| APLN | CLEC4F |
| APOA1 | CNDP1 |
| AQP9 | COL25A1 |
| ARAP3 | COLEC11 |
| ARFRP1 | CPAMD8 |
| ARHGAP6 | CRYM |
| ARHGEF10 | CSHL1 |
| ARHGEF2 | CTAG1A |
| ARID4A | CX3CL1 |
| ARL13B | CYP2C18 |
| ARL4C | CYP2C9 |
| ARL6IP6 | DCD |
| ARMCX1 | DDX4 |
| ARNTL | DLX6 |
| ASMT | DNASE1 |
| ASPA | DPPA2 |
| ASPRV1 | DPYSL5 |
| ATAT1 | DSEL |
| ATOH8 | EEF1A2 |
| ATP11B | ENTPD5 |
| AXIN2 | EPS8 |
| AXL | ERI2 |
| BAG2 | ERVMER34-1 |
| BAZ2B | F2 |
| BBS9 | FAM167A-AS1 |
| BCL2L2 | FAM3D |
| BDKRB1 | FAXDC2 |
| BEND6 | FBXO27 |
| BEX2 | FCN2 |
| BHLHE41 | FIGLA |
| BMI1 | FKBP5 |
| BMP15 | FOXC1 |
| BMP2K | FXYD2 |
| BMP4 | G6PC |
| BNC2 | GAL3ST1 |
| BRD4 | GALNT4 |
| BST2 | GDA |
| BTG3 | GFRA4 |
| BTN1A1 | GJC3 |
| C10orf11 | GLYATL2 |
| C11orf45 | GPR68 |
| C11orf63 | GPR78 |
| C19orf12 | GSG1L |
| C1QL4 | GUCA2B |
| C1QTNF2 | HECW2 |
| C1QTNF3 | HGD |
| C1QTNF5 | HOOK3 |
| C1QTNF5///MFRP | HORMAD2 |
| C1R | HOXC11 |
| C1S | HSD17B2 |
| C22orf23 | HSPB9 |
| C3 | IDO1 |
| C5orf46 | IGFBP1 |
| C8orf58 | IGSF1 |
| CALD1 | IL17C |
| CALHM2 | IL22RA1 |
| CASD1 | IL25 |
| CAV1 | IPMK |
| CCDC102B | KCNG2 |
| CCDC3 | KCNQ2 |
| CCDC6 | KLB |
| CCDC80 | KRT5 |
| CCDC82 | KSR1 |
| CCDC88A | LCN2 |
| CCL11 | LEFTY1 |
| CCNA2 | LINC00588 |
| CCND2 | LIPG |
| CCNT2 | LNX1 |
| CD109 | LRRC19 |
| CD200R1 | LY6D |
| CD86 | MAGEA6 |
| CD99 | MAGEB2 |
| CDCA7L | MAP2K5 |
| CDH11 | MAP2K6 |
| CDH2 | MC4R |
| CDH23 | MGAT5 |
| CDK14 | MOGAT1 |
| CENPQ | MUC13 |
| CEP120 | MUC2 |
| CEP170 | MUC7 |
| CEP41 | MYH6 |
| CFL2 | MYO1A |
| CGREF1 | NAPSA |
| CHD9 | NCOA7 |
| CHEK1 | NKAIN4 |
| CHL1 | NKX2-8 |
| CHN1 | NKX6-2 |
| CHRM3 | NLGN4Y |
| CHRNA1 | NMNAT2 |
| CHSY3 | NOS1 |
| CIART | NOS2 |
| CKLF | NRXN3 |
| CKS1B | OR10K2 |
| CLDN16 | OR3A3 |
| CLDN18 | OTP |
| CLEC2B | OTX1 |
| CLEC4A | PAGE1 |
| CLGN | PCSK9 |
| CLIC2 | PDE11A |
| CLIP3 | PDE7B |
| CLMP | PDZK1IP1 |
| CLN5 | PIGR |
| CLN8 | PIK3R3 |
| CNN1 | PITX1 |
| CNOT9 | PKP1 |
| CNRIP1 | PLA2G10 |
| COL10A1 | PLA2G4F |
| COL11A1 | POU3F1 |
| COL12A1 | PRODH |
| COL16A1 | PROM1 |
| COL1A1 | RBP4 |
| COL1A2 | RDH12 |
| COL3A1 | RGS6 |
| COL5A1 | RHOV |
| COL5A2 | RHOXF1 |
| COL6A1 | RLBP1 |
| COL6A2 | RNF186 |
| COL6A3 | RPRD1B |
| COL8A2 | RXFP1 |
| COPZ2 | S100P |
| CORIN | SAA1 |
| COX7A1 | SAA2 |
| CPE | SAA4 |
| CPQ | SCG3 |
| CPTP | SCNN1G |
| CRABP2 | SDE2 |
| CRADD | SEMA4G |
| CREB3L3 | SEMA6A |
| CRISPLD1 | SFN |
| CROT | SFTA2 |
| CRYAB | SIM1 |
| CSGALNACT2 | SIX6 |
| CT47A11 | SLC18A1 |
| CTGF | SLC38A3 |
| CTHRC1 | SLC3A1 |
| CTSK | SLC44A3 |
| CUTC | SLC6A20 |
| CXCL12 | SMPD3 |
| CXCR4 | SPATA13 |
| CXorf56 | SPTB |
| CXorf57 | STYK1 |
| CYB5D1 | SYBU |
| CYP11B2 | TAS2R38 |
| CYP27A1 | TFAP2B |
| CYP2U1 | TFCP2L1 |
| CYP7B1 | TGM3 |
| DAB2 | TGM7 |
| DACH1 | TLX3 |
| DACH2 | TMEM56 |
| DACT1 | TMIGD2 |
| DACT3 | TRIM51 |
| DAPL1 | TUBAL3 |
| DAW1 | USP43 |
| DAZL | VAX2 |
| DCAKD | VGF |
| DCHS1 | VSTM2A |
| DCN | WDR87 |
| DDR2 | XAGE2 |
| DEPDC7 | XAGE3 |
| DES | ZPBP |
| DFNA5 |  |
| DGKB |  |
| DHDDS |  |
| DIDO1 |  |
| DIEXF |  |
| DKK2 |  |
| DKK3 |  |
| DLGAP1-AS2 | |
| DLL4 |  |
| DMRTB1 |  |
| DNAJB4 |  |
| DNAJC27 |  |
| DOCK4 |  |
| DOK5 |  |
| DPAGT1 |  |
| DPT |  |
| DPYSL3 |  |
| DSC3 |  |
| DTNA |  |
| DUSP11 |  |
| DYNC2H1 |  |
| DZIP1 |  |
| DZIP3 |  |
| ECM1 |  |
| EDEM3 |  |
| EDIL3 |  |
| EDNRA |  |
| EDNRB |  |
| EDRF1 |  |
| EFEMP2 |  |
| EFHB |  |
| EFNB2 |  |
| EGFL6 |  |
| EGR2 |  |
| EIF5A2 |  |
| ELN |  |
| EMILIN1 |  |
| EMX2 |  |
| ENOX2 |  |
| ENPP1 |  |
| EPB41L3 |  |
| EPHA4 |  |
| EPHB6 |  |
| EPM2A |  |
| ERG |  |
| ERO1B |  |
| ERRFI1 |  |
| ETV1 |  |
| EVA1C |  |
| EXTL2 |  |
| F10 |  |
| FAM110B |  |
| FAM118B |  |
| FAM122B |  |
| FAM126A |  |
| FAM175A |  |
| FAM178B |  |
| FAM181A |  |
| FAM182B |  |
| FAM198B |  |
| FAM227A |  |
| FAM50B |  |
| FAM76B |  |
| FAM83D |  |
| FAM9C |  |
| FAP |  |
| FAT2 |  |
| FBLN1 |  |
| FBN1 |  |
| FBXL3 |  |
| FBXL7 |  |
| FBXO32 |  |
| FCGR2B |  |
| FEM1B |  |
| FEM1C |  |
| FEZ1 |  |
| FEZF1 |  |
| FGFBP2 |  |
| FGFRL1 |  |
| FGGY |  |
| FHL1 |  |
| FIGN |  |
| FIGNL1 |  |
| FILIP1L |  |
| FLI1 |  |
| FLJ30679 |  |
| FLNC |  |
| FLRT2 |  |
| FMNL1 |  |
| FMOD |  |
| FN1 |  |
| FN3K |  |
| FNDC5 |  |
| FOXA2 |  |
| FOXP4 |  |
| FPR3 |  |
| FRAS1 |  |
| FST |  |
| FSTL1 |  |
| FSTL3 |  |
| FUNDC2 |  |
| FXYD1 |  |
| FXYD5 |  |
| FYN |  |
| FZD4 |  |
| FZD6 |  |
| FZD7 |  |
| GAB2 |  |
| GABRR1 |  |
| GALNT13 |  |
| GAS1 |  |
| GAS2L3 |  |
| GAS6 |  |
| GATA6 |  |
| GCKR |  |
| GEM |  |
| GFOD1 |  |
| GFRA1 |  |
| GJA4 |  |
| GJA5 |  |
| GJB2 |  |
| GJC2 |  |
| GLT8D2 |  |
| GM2A |  |
| GMCL1 |  |
| GNA13 |  |
| GNA15 |  |
| GNB1 |  |
| GNG12 |  |
| GNG7 |  |
| GP1BA |  |
| GP5 |  |
| GPR137B |  |
| GPR176 |  |
| GPR183 |  |
| GPR19 |  |
| GPR34 |  |
| GPRASP2 |  |
| GPX8 |  |
| GRB10 |  |
| GREM1 |  |
| GRPR |  |
| GSN |  |
| GSTT2 |  |
| GTF2IRD2 |  |
| GYPC |  |
| H2AFY2 |  |
| HACD4 |  |
| HAND2 |  |
| HAS1 |  |
| HAS2 |  |
| HAVCR1 |  |
| HBB |  |
| HCN4 |  |
| HEG1 |  |
| HEPH |  |
| HEYL |  |
| HHEX |  |
| HIC1 |  |
| HIGD1B |  |
| HIKESHI |  |
| HIST1H4G |  |
| HMGN5 |  |
| HOPX |  |
| HRK |  |
| HSD17B11 |  |
| HSD17B14 |  |
| HSD3B1 |  |
| HSF2BP |  |
| HSPA12B |  |
| HSPA13 |  |
| HSPA6 |  |
| HSPB2 |  |
| HSPB8 |  |
| HTR2C |  |
| HTRA3 |  |
| ID3 |  |
| IFT46 |  |
| IGDCC4 |  |
| IGF1 |  |
| IKZF4 |  |
| IL16 |  |
| IL20 |  |
| IL34 |  |
| IMPA1 |  |
| INHBA |  |
| IRF8 |  |
| ISLR |  |
| ITGA1 |  |
| ITGA11 |  |
| ITGAV |  |
| ITGB1BP1 |  |
| ITGB5 |  |
| ITPR1 |  |
| JAM2 |  |
| JAM3 |  |
| JPH2 |  |
| KANK2 |  |
| KCNAB1 |  |
| KCNE4 |  |
| KCNF1 |  |
| KCNH8 |  |
| KCNJ8 |  |
| KCNN3 |  |
| KCTD12 |  |
| KDELC1 |  |
| KIAA0226L | |
| KIAA1429 |  |
| KIAA1524 |  |
| KIF1B |  |
| KIF26B |  |
| KIF3C |  |
| KIFAP3 |  |
| KLF12 |  |
| KLF2 |  |
| KLHL6 |  |
| KPNA3 |  |
| KRT14 |  |
| KRT2 |  |
| KRTAP9-9 |  |
| L3MBTL1 |  |
| LAIR2 |  |
| LAYN |  |
| LCT |  |
| LDB2 |  |
| LFNG |  |
| LGALS1 |  |
| LGALS2 |  |
| LGR6 |  |
| LHFP |  |
| LHFPL1 |  |
| LHX4 |  |
| LIG4 |  |
| LIMS2 |  |
| LINC00260 | |
| LINC00526 | |
| LINGO2 |  |
| LIX1L |  |
| LMCD1 |  |
| LOC100128482 | |
| LOC100130428 | |
| LOC643733 | |
| LOXL1 |  |
| LOXL2 |  |
| LRIG1 |  |
| LRP2 |  |
| LRRC14 |  |
| LRRC34 |  |
| LRRTM1 |  |
| LRTOMT |  |
| LTBP2 |  |
| LTBP4 |  |
| LUM |  |
| LXN |  |
| LYPD1 |  |
| MAD2L1 |  |
| MAEL |  |
| MAF |  |
| MAFB |  |
| MAGEH1 |  |
| MAP7D3 |  |
| MAPK8 |  |
| MAPK8IP1 |  |
| MARCKS |  |
| MARCO |  |
| MAT2B |  |
| MATN2 |  |
| MB21D2 |  |
| MBD3L1 |  |
| MBNL2 |  |
| ME3 |  |
| MEDAG |  |
| MEF2C |  |
| MEI1 |  |
| MEIOB |  |
| MEIS2 |  |
| METRNL |  |
| METTL10 |  |
| METTL16 |  |
| MFAP2 |  |
| MFAP4 |  |
| MFN1 |  |
| MGC24103 |  |
| MICA |  |
| MILR1 |  |
| MKL1 |  |
| MKRN3 |  |
| MLLT10 |  |
| MMD |  |
| MMP11 |  |
| MMP16 |  |
| MMP19 |  |
| MMP2 |  |
| MMP23B |  |
| MMRN1 |  |
| MN1 |  |
| MORC1 |  |
| MOSPD2 |  |
| MPDZ |  |
| MPP4 |  |
| MPP6 |  |
| MRGPRF |  |
| MRGPRX2 |  |
| MRVI1 |  |
| MS4A1 |  |
| MS4A7 |  |
| MSANTD2 |  |
| MTDH |  |
| MXRA8 |  |
| MYADM |  |
| MYBL1 |  |
| MYL9 |  |
| MYO1B |  |
| MYOT |  |
| N4BP2L1 |  |
| NAALADL1 |  |
| NAIP |  |
| NAP1L3 |  |
| NAPB |  |
| NBL1 |  |
| NBPF14 |  |
| NCF2 |  |
| NCR2 |  |
| NDN |  |
| NEGR1 |  |
| NEIL3 |  |
| NEK1 |  |
| NEK7 |  |
| NES |  |
| NEU3 |  |
| NEUROD1 |  |
| NEUROD4 |  |
| NFIA |  |
| NFIB |  |
| NID2 |  |
| NKAIN2 |  |
| NKD1 |  |
| NKIRAS1 |  |
| NLRC4 |  |
| NLRP7 |  |
| NMT2 |  |
| NOX4 |  |
| NPAS3 |  |
| NPAT |  |
| NPHP1 |  |
| NPR2 |  |
| NPTN |  |
| NR1D2 |  |
| NR2F1 |  |
| NR3C1 |  |
| NR4A2 |  |
| NREP |  |
| NRP2 |  |
| NTM |  |
| NUFIP1 |  |
| NXPE3 |  |
| OAT |  |
| ODF3 |  |
| OLFML2B |  |
| OLFML3 |  |
| OPN1MW |  |
| OR11G2 |  |
| OR13C4 |  |
| OR1A1 |  |
| OR1J4 |  |
| OR1S1 |  |
| OR2T10 |  |
| OR5D14 |  |
| OR5P2 |  |
| OSM |  |
| OTUD6B-AS1 | |
| OXCT1 |  |
| OXTR |  |
| P2RY10 |  |
| PACRGL |  |
| PALLD |  |
| PALM |  |
| PALMD |  |
| PAM |  |
| PAQR3 |  |
| PARG |  |
| PARL |  |
| PARP15 |  |
| PART1 |  |
| PCDH18 |  |
| PCDHB10 |  |
| PCOLCE |  |
| PCOLCE2 |  |
| PDGFD |  |
| PDGFRA |  |
| PDGFRB |  |
| PDGFRL |  |
| PDIA5 |  |
| PDLIM2 |  |
| PDLIM3 |  |
| PDPN |  |
| PEA15 |  |
| PECAM1 |  |
| PER3 |  |
| PEX2 |  |
| PGAP1 |  |
| PGBD1 |  |
| PHF1 |  |
| PHF14 |  |
| PHLDB1 |  |
| PHLDB2 |  |
| PHTF2 |  |
| PICALM |  |
| PIEZO2 |  |
| PIN4 |  |
| PITPNM3 |  |
| PKIA |  |
| PLA2G4A |  |
| PLAU |  |
| PLAUR |  |
| PLCB2 |  |
| PLCXD1 |  |
| PLIN2 |  |
| PLOD2 |  |
| PLPPR4 |  |
| PLSCR4 |  |
| PLXNA2 |  |
| PMP22 |  |
| PNLIPRP2 |  |
| PNMA2 |  |
| PNMA3 |  |
| PNOC |  |
| PODN |  |
| POM121 |  |
| POM121L9P | |
| POSTN |  |
| POU4F3 |  |
| PPFIA2 |  |
| PPP1R12A |  |
| PPP1R14A |  |
| PPP2R2B |  |
| PPP3CC |  |
| PRDM2 |  |
| PRDX3 |  |
| PRELP |  |
| PRNP |  |
| PROCR |  |
| PROS1 |  |
| PRPS1 |  |
| PRSS23 |  |
| PSAP |  |
| PSMA8 |  |
| PTGDS |  |
| PTGER4 |  |
| PTN |  |
| PTPN20 |  |
| PTPRG |  |
| PTRF |  |
| QKI |  |
| RAB11FIP2 | |
| RAB28 |  |
| RAB31 |  |
| RAB32 |  |
| RAB33A |  |
| RADIL |  |
| RANBP6 |  |
| RAP1GDS1 |  |
| RASGRF2 |  |
| RASSF2 |  |
| RBM11 |  |
| RBM38 |  |
| RBM7 |  |
| RDX |  |
| RECK |  |
| RECQL5 |  |
| REG3G |  |
| REXO2 |  |
| RFXAP |  |
| RGCC |  |
| RGS1 |  |
| RGS16 |  |
| RGS2 |  |
| RGS22 |  |
| RGS4 |  |
| RGS5 |  |
| RIMS3 |  |
| RIN2 |  |
| RIN3 |  |
| RNASE3 |  |
| RND3 |  |
| RNF32 |  |
| ROBO2 |  |
| ROBO3 |  |
| ROR1 |  |
| RORA |  |
| RPL23AP32 | |
| RPS4Y1 |  |
| RPS6KA3 |  |
| RPTN |  |
| RRN3P1 |  |
| RUNX1T1 |  |
| RUSC1-AS1 | |
| S100A2 |  |
| S100A3 |  |
| S1PR1 |  |
| SACS |  |
| SARAF |  |
| SATB1 |  |
| SCN2B |  |
| SCN4B |  |
| SDHAF3 |  |
| SDHD |  |
| SEC23A |  |
| SEC24D |  |
| SELM |  |
| SELT |  |
| SEMA3A |  |
| SEMA3E |  |
| SEMA3F |  |
| SERPINB6 |  |
| SERPIND1 |  |
| SERPINE2 |  |
| SERPINF1 |  |
| SESN3 |  |
| SETD7 |  |
| SEZ6L |  |
| SFRP2 |  |
| SFRP4 |  |
| SGIP1 |  |
| SH3BGRL |  |
| SH3D19 |  |
| SH3PXD2B |  |
| SI |  |
| SKAP2 |  |
| SLC16A12 |  |
| SLC1A1 |  |
| SLC23A2 |  |
| SLC25A24 |  |
| SLC25A27 |  |
| SLC29A3 |  |
| SLC2A12 |  |
| SLC2A5 |  |
| SLC2A9 |  |
| SLC30A8 |  |
| SLC34A1 |  |
| SLC35B3 |  |
| SLC39A14 |  |
| SLC46A2 |  |
| SLC4A5 |  |
| SLIT2 |  |
| SMAD4 |  |
| SMAD7 |  |
| SMAP1 |  |
| SMIM3 |  |
| SMPDL3A |  |
| SMURF2 |  |
| SNAI2 |  |
| SNCG |  |
| SNIP1 |  |
| SNX16 |  |
| SNX18 |  |
| SNX19 |  |
| SOCS5 |  |
| SOGA1 |  |
| SPARC |  |
| SPARCL1 |  |
| SPATA8 |  |
| SPOCK2 |  |
| SPON2 |  |
| SPRED1 |  |
| SRGN |  |
| SRI |  |
| SRP9 |  |
| SRPRA |  |
| SRPX |  |
| SRPX2 |  |
| SRSF8 |  |
| SSPN |  |
| SSTR5 |  |
| SSUH2 |  |
| SSX5 |  |
| ST3GAL1 |  |
| ST3GAL5 |  |
| ST6GALNAC2 | |
| ST6GALNAC5 | |
| STAMBPL1 |  |
| STEAP1 |  |
| STRN |  |
| STXBP5 |  |
| SULF1 |  |
| SYNC |  |
| SYNDIG1 |  |
| SYNE1 |  |
| SYNPO |  |
| TAF1A |  |
| TAF2 |  |
| TAGLN |  |
| TAS2R7 |  |
| TBC1D8B |  |
| TBPL1 |  |
| TBRG1 |  |
| TBX2 |  |
| TBX3 |  |
| TCEAL7 |  |
| TCF21 |  |
| TCF4 |  |
| TCFL5 |  |
| TDO2 |  |
| TEK |  |
| TENM3 |  |
| TEX29 |  |
| TFEC |  |
| TGFB1I1 |  |
| TGFB3 |  |
| TGFBI |  |
| TGM1 |  |
| THBD |  |
| THBS1 |  |
| THBS2 |  |
| THNSL1 |  |
| THY1 |  |
| THYN1 |  |
| TIAM2 |  |
| TIMP3 |  |
| TLE4 |  |
| TLR1 |  |
| TM4SF1 |  |
| TMC2 |  |
| TMEM135 |  |
| TMEM136 |  |
| TMEM14A |  |
| TMEM158 |  |
| TMEM182 |  |
| TMEM190 |  |
| TMEM200A |  |
| TMEM204 |  |
| TMEM217 |  |
| TMEM243 |  |
| TMEM35A |  |
| TMEM45A |  |
| TMEM55A |  |
| TMEM99 |  |
| TMPO |  |
| TMPRSS11A | |
| TMPRSS11D | |
| TMPRSS5 |  |
| TMPRSS6 |  |
| TNC |  |
| TNFRSF8 |  |
| TNFSF4 |  |
| TOX |  |
| TP53AIP1 |  |
| TPM4 |  |
| TRAM1L1 |  |
| TRIM37 |  |
| TRIM58 |  |
| TRNT1 |  |
| TRO |  |
| TRPC3 |  |
| TRPC5 |  |
| TRPC6 |  |
| TRPV3 |  |
| TSC1 |  |
| TSC22D3 |  |
| TSHZ3 |  |
| TSPAN19 |  |
| TSPYL5 |  |
| TTC28 |  |
| TTL |  |
| TTR |  |
| TUBB2A |  |
| TUBE1 |  |
| TXNRD1 |  |
| UBA3 |  |
| UBE2E2 |  |
| UBE2J2 |  |
| UGT2A3 |  |
| UNC5B |  |
| VAMP3 |  |
| VAMP4 |  |
| VCAM1 |  |
| VEGFC |  |
| VEZF1 |  |
| VGLL3 |  |
| VIM |  |
| VPS26A |  |
| VPS50 |  |
| WDFY1 |  |
| WISP1 |  |
| WNT11 |  |
| WNT2 |  |
| WRB |  |
| WT1 |  |
| XPR1 |  |
| YTHDF3 |  |
| ZBED2 |  |
| ZBED8 |  |
| ZC2HC1A |  |
| ZCCHC24 |  |
| ZCCHC3 |  |
| ZDHHC2 |  |
| ZEB1 |  |
| ZEB2 |  |
| ZFAND1 |  |
| ZFP42 |  |
| ZFPM2 |  |
| ZIC5 |  |
| ZMAT3 |  |
| ZMIZ1 |  |
| ZMYM6 |  |
| ZNF175 |  |
| ZNF202 |  |
| ZNF25 |  |
| ZNF285 |  |
| ZNF300 |  |
| ZNF318 |  |
| ZNF404 |  |
| ZNF449 |  |
| ZNF454 |  |
| ZNF461 |  |
| ZNF521 |  |
| ZNF550 |  |
| ZNF551 |  |
| ZNF556 |  |
| ZNF559 |  |
| ZNF608 |  |
| ZNF684 |  |
| ZNF75D |  |
| ZNF771 |  |
| ZSWIM1 |  |
