# Supplementary material for: Dysregulation of pseudogene/lncRNA-hsa-miR-363-3p-SPOCK2 pathway fuels stage progression of ovarian cancer
Source: Aging (Albany NY). 2019 Dec 3;11(23):11416–39. doi: 10.18632/aging.102538 (PMC6932902; doi:10.18632/aging.102538)
Supplement: Supplementary Table 3 [file aging-11-102538-s002..docx]

**Supplementary Table 3. The co-expressed genes of SPOCK2 from UALCAN and GEPIA databases.**

| Co-expressed genes determined by UALCAN | Co-expressed genes determined by GEPIA |
| --- | --- |
| RASGRP4 | KCNB1 |
| SLC48A1 | RASGRP4 |
| ADORA1 | UNC5B |
| UNC5B | SLC48A1 |
| OXTR | RP11-150D20.5 |
| CRB2 | TARM1 |
| TRIM36 | RP11-57H12.5 |
| CADM3 | OR9G1 |
| SLC22A18AS | CRB2 |
| ZBED2 | C10orf53 |
| TNS3 | ZBED2 |
| BEAN | LINC01343 |
| KIF21A | OXTR |
| S100A10 | HIGD1AP5 |
| CLIC5 | RP3-468B3.2 |
| CAMK2G | RP11-456O19.5 |
| RAB19 | ADORA1 |
| CACNG4 | TNS3 |
| TBC1D2 | EIF2B5-AS1 |
| FAM70A | MAGEB3 |
| UPK3B | RP11-506B6.7 |
| MYADM | CT45A8 |
| ANXA7 | RP11-603B24.6 |
| CLDN15 | TRIM36 |
| WNT10A | CT45A9 |
| CCDC85A | CAMK2G |
| CD151 | RAB19 |
| CASKIN2 | ANXA2 |
| OBP2B | CADM3 |
| AMOTL2 | FAM180B |
| DTX4 | AC007743.1 |
| IGFBP6 | CLIC5 |
| ANXA2P2 | CTC-459F4.5 |
| ST6GAL2 | TBC1D2 |
| ANXA2 | AMOTL2 |
| KRT80 | KIF21A |
| CLSTN2 | S100A10 |
| ANXA9 | GRM2 |
| DNM3 | TMEM255A |
| SHISA4 | MYADM |
| PLA2G7 | B3GALT2 |
| INF2 | CHRDL1 |
| CHRDL1 | DNM3 |
| RAPGEF3 | ZNF385B |
| SLC4A11 | CACNG4 |
| FRMD5 | WNT10A |
| PNPLA2 | UPK3B |
| RNASEL | SLC22A18AS |
| TPRN | DTX4 |
| OLFML2A | DNAJC5G |
| TGM1 | CTD-2265O21.7 |
| VSIG10L | CDK15 |
| SERPINB5 | CLDN15 |
| LRRN4 | RAPGEF3 |
| FNDC4 | Metazoa_SRP |
| PRSS33 | ANXA7 |
| ST5 | KRT80 |
| C12orf61 | CCDC85A |
| GNG12 | SLC6A3 |
| NGFR | CTIF |
| VSTM2L | BCL2L1 |
| ZNF488 | CD151 |
| CTAGE5 | ASIC2 |
| TNNT2 | RP11-166B2.8 |
| BAIAP2 | SHISA4 |
| TMEM150C | CH17-360D5.2 |
| SPTAN1 | CLSTN2 |
| ARL13B | MAGI2-AS3 |
| SERPINA5 | LINC01465 |
| ZMIZ1 | FNDC4 |
| C9orf3 | TGM1 |
| PHOX2A | ITGA3 |
| CHST11 | ARL13B |
| SLC29A3 | CASKIN2 |
| LMO1 | SLC29A3 |
| ANO9 | BAIAP2 |
| PKP3 | ANXA9 |
| LEPREL1 | C15orf52 |
| VCL | ANXA2P2 |
| DENND1A | PSAP |
| ARNTL | HPX |
| BET1L | KCNV1 |
| TMEM9B | ZMIZ1 |
| GDPD5 | TPRN |
| FST | RP5-875H18.9 |
| DUSP8 | GPR45 |
| SCD5 | RNASEL |
| FAM69A | VSTM2L |
| PLCD3 | AJAP1 |
| LOC158376 | LRRN4 |
| MRC2 | FSTL3 |
| TNS1 | RN7SL343P |
| ARPC1B | RN7SL640P |
| SMPD1 | RN7SL462P |
| PXN | RN7SL422P |
| RHOF | RP11-34A14.3 |
| CX3CL1 | RP11-472F21.1 |
| RIC8A | BET1L |
|  | RP11-583F24.3 |
|  | ST5 |
|  | RP11-583F24.4 |
|  | OLFML2A |
|  | PXN |
|  | RHOF |
|  | RP11-208K4.1 |
|  | BEAN1 |
|  | FSCN2 |
|  | SLC4A11 |
|  | INF2 |
|  | CTC-360P9.1 |
|  | NCOR2 |
|  | CHST11 |
|  | RP11-337L12.1 |
|  | FAM138A |
|  | UNC5B-AS1 |
|  | VCL |
|  | CACNG1 |
|  | NKX2-8 |
|  | RP11-398E10.1 |
|  | RNA5SP392 |
|  | IGFBP6 |
|  | HFM1 |
|  | BPIFA2 |
|  | SLC13A2 |
|  | RIC8A |
|  | ST6GAL2 |
|  | APOOP4 |
|  | MYOF |
|  | RP1-313L4.3 |
|  | PLA2G7 |
|  | PPL |
|  | CALCOCO1 |
|  | SCRG1 |
|  | AC003973.5 |
|  | LINC01105 |
|  | RP11-482H16.1 |
|  | GNG12 |
|  | WSCD2 |
|  | SCD5 |
|  | AHNAK |
|  | TNS1 |
|  | PNPLA2 |
|  | PRSS33 |
|  | SERPINB5 |
|  | SMPD1 |
|  | SORBS1 |
|  | NCS1 |
|  | TPP1 |
|  | AC017104.6 |
|  | RP11-490M8.1 |
|  | RP11-23P13.6 |
|  | VSIG10L |
|  | TUBB6 |
|  | SSX6 |
|  | LINC00920 |
|  | EXTL1 |
|  | PDLIM2 |
|  | CLEC4M |
|  | BLCAP |
|  | RP11-307F22.2 |
|  | ARNTL |
|  | SLC25A22 |
|  | RP11-483I24.1 |
|  | PLEKHA7 |
|  | PTRF |
|  | ARPC1B |
|  | AKR1C8P |
|  | RP11-567M16.2 |
|  | SERPINA5 |
|  | LINC01304 |
|  | ANO9 |
|  | CRIM1 |
|  | FAM69A |
|  | RARG |
|  | CTD-2194D22.1 |
|  | GDPD5 |
|  | EHD1 |
|  | EPHA2 |
|  | RP5-857K21.7 |
|  | NBL1 |
|  | TMEM9B |
|  | BCAR1 |
|  | SEC24C |
|  | CTD-2128A3.2 |
|  | TGM2 |
|  | TMEM120B |
|  | CTD-2151A2.1 |
|  | LAYN |
|  | RP1-137K24.1 |
|  | HAR1B |
|  | CH507-236L23.5 |
|  | FAM65A |
|  | SFXN3 |
|  | TFG |
|  | TNNT2 |
|  | RP11-38L15.2 |
|  | NACC2 |
|  | PKP3 |
|  | CELSR2 |
|  | ZNF365 |
