# Supplementary material for: Dysregulation of pseudogene/lncRNA-hsa-miR-363-3p-SPOCK2 pathway fuels stage progression of ovarian cancer
Source: Aging (Albany NY). 2019 Dec 3;11(23):11416–39. doi: 10.18632/aging.102538 (PMC6932902; doi:10.18632/aging.102538)
Supplement: Supplementary Tables 4 and 5 [file aging-11-102538-s003..pdf]

Supplementary Table 4. The co-expressed genes of SPOCK2 commonly appeared in UALCAN and GEPIA databases.

Co-expressed genes
RASGRP4
SLC48A1
ADORA1
UNC5B
OXTR
CRB2
TRIM36
CADM3
SLC22A18AS
ZBED2
TNS3
KIF21A
S100A10
CLIC5
CAMK2G
RAB19
CACNG4
TBC1D2
UPK3B
MYADM
ANXA7
CLDN15
WNT10A
CCDC85A
CD151
CASKIN2
AMOTL2
DTX4
IGFBP6
ANXA2P2
ST6GAL2
ANXA2
KRT80
CLSTN2
ANXA9
DNM3
SHISA4
PLA2G7
INF2
CHRDL1
RAPGEF3
SLC4A11
PNPLA2
RNASEL

TPRN
OLFML2A
TGM1
VSIG10L
SERPINB5
LRRN4
FNDC4
PRSS33
ST5
GNG12
VSTM2L
TNNT2
BAIAP2
ARL13B
SERPINA5
ZMIZ1
CHST11
SLC29A3
ANO9
PKP3
VCL
ARNTL
BET1L
TMEM9B
GDPD5
SCD5
FAM69A
TNS1
ARPC1B
SMPD1
PXN
RHOF
RIC8A

Supplementary Table 5. The potential upstream lncRNAs of hsa-miR-363-3p predicted by starBase and miRNet databases.

lncRNAs predicted by starBase	lncRNAs predicted by miRNet
LINC01128	NEFL
MDS2	XIST
AC239868.3	SCAMP1
AL358472.2	MALAT1
LINC00467	SNHG5
C1orf143	OIP5-AS1
AC074117.1	RNU4ATAC
AC016700.3	DAPK1-IT1
PAX8-AS1	AC005307.3
AC018470.1	AC005943.5
AC016708.1	AC007228.9
AC105760.2	AC034220.3
WWTR1-IT1	AC074117.10
AC104472.1	AC093627.10
AC007620.3	LINC00657
CTBP1-AS2	PITPNA-AS1
AC097376.2	RP1-5O6.6
AC104793.1	RP11-206L10.11
PURPL	RP11-234O6.2
NR2F1-AS1	RP11-449D8.1
MIR3936HG	RP11-46C20.1
SNHG4	RP11-473I1.10
AL049555.1	RP11-492E3.1
SNHG5	RP11-65F13.2
AL513550.1	RP11-815I9.4
AL022069.1	RP11-98I9.4
AC093627.4	RP3-341D10.4
AC011294.1	RP3-368A4.6
FEZF1-AS1	RP3-508I15.9
WEE2-AS1	RP3-523K23.2
AC021242.3	RP4-714D9.5
AF131215.6	SNHG14
AC124067.4	TBX5-AS1
AC084082.1	
AL354707.1	
DAPK1-IT1	
LINC00963	
AL117339.4	
LINC00858	
KCNQ1OT1	
MALAT1	
AP000577.1	
AP001541.1	
TBX5-AS1	

HNF1A-AS1
AC131212.3
LINC00365
INTS6-AS1
AC005519.1
AL136040.1
LINC02321
LINC01550
MEG8
PWAR5
SNHG14
ARHGAP11B
OIP5-AS1
PLA2G4E-AS1
AC090510.3
GABPB1-IT1
GABPB1-AS1
AC055855.1
AC015712.2
AC130650.2
PITPNA-AS1
AC026271.3
CCDC144NL-AS1
AC111170.3
AC087741.1
LINC01915
AC011825.4
AC018445.3
AC011447.7
AC005394.2
AC008555.8
AC012617.1
AC022150.4
AC007228.2
AC012313.1
NORAD
SNHG17
DUXAP8
AP000553.1
AL022322.1
AL021707.2
NDUFA6-AS1
LINC01560
XIST
JPX
Z83843.1
